# ETV3 and ETV6 enable monocyte differentiation into dendritic cells by repressing macrophage fate commitment

**DOI:** 10.1038/s41590-022-01374-0

**Published:** 2022-12-21

**Authors:** Javiera Villar, Adeline Cros, Alba De Juan, Lamine Alaoui, Pierre-Emmanuel Bonte, Colleen M. Lau, Ioanna Tiniakou, Boris Reizis, Elodie Segura

**Affiliations:** 1 Institut Curie, PSL Research University, INSERM, U932,, Paris, France; 2 Department of Pathology, New York University Grossman School of Medicine, New York, NY USA

**Keywords:** Monocytes and macrophages, Gene regulation in immune cells

## Abstract

In inflamed tissues, monocytes differentiate into macrophages (mo-Macs) or dendritic cells (mo-DCs). In chronic nonresolving inflammation, mo-DCs are major drivers of pathogenic events. Manipulating monocyte differentiation would therefore be an attractive therapeutic strategy. However, how the balance of mo-DC versus mo-Mac fate commitment is regulated is not clear. In the present study, we show that the transcriptional repressors ETV3 and ETV6 control human monocyte differentiation into mo-DCs. ETV3 and ETV6 inhibit interferon (IFN)-stimulated genes; however, their action on monocyte differentiation is independent of IFN signaling. Instead, we find that ETV3 and ETV6 directly repress mo-Mac development by controlling *MAFB* expression. Mice deficient for Etv6 in monocytes have spontaneous expression of IFN-stimulated genes, confirming that Etv6 regulates IFN responses in vivo. Furthermore, these mice have impaired mo-DC differentiation during inflammation and reduced pathology in an experimental autoimmune encephalomyelitis model. These findings provide information about the molecular control of monocyte fate decision and identify ETV6 as a therapeutic target to redirect monocyte differentiation in inflammatory disorders.

## Abstract (web summary)

The factors controlling monocyte fate commitment toward macrophages versus dendritic cells are unclear. Here the authors show that ETV3 and ETV6 enable dendritic cell differentiation by repressing the macrophage transcriptional program.

## Main

Monocytes and monocyte-derived cells are central players in the initiation and resolution of inflammatory responses. In chronic inflammatory diseases, monocyte-derived antigen-presenting cells (APCs) become major drivers of the physiopathology by stimulating pathogenic T cells. Blocking monocyte differentiation therefore represents an attractive therapeutic strategy. A major hurdle is the paucity of molecular targets, due to limited knowledge of the molecular regulation of monocyte fate commitment.

Circulating monocytes infiltrate mucosal or inflamed tissues where they differentiate into mo-Macs or mo-DCs^1–3^. Mo-Macs are generally involved in homeostasis and tissue repair, whereas mo-DCs present antigens to T cells directly in tissues. However, in chronic nonresolving inflammation, T cell stimulation by mo-DCs becomes deleterious. In Crohn’s disease, rheumatoid arthritis and psoriasis, mo-DCs secrete high amounts of interleukin (IL)-23 and stimulate helper type 17 T cells (T_H_17) cells, two major drivers of the physiopathology^4–7^. In mouse models, mo-DCs induce pathogenic T cells that mediate tissue damage in experimental autoimmune encephalomyelitis (EAE)^8^ and colitis^9,10^. Blocking monocyte differentiation has therefore emerged as a potential therapeutic strategy for inflammatory disorders. Pharmacological inhibition of monocyte recruitment suppresses the development of colitis^11^ and the severity of EAE^12^. Inducing monocyte apoptosis with nanoparticles reduces inflammation and disease symptoms in colitis, EAE, peritonitis and virus-induced encephalitis^13^. Finally, impairing monocyte survival and differentiation via macrophage–colony-stimulating factor (M-CSF) receptor blockade reduces inflammation in arthritis^14,15^. However, a major caveat of these approaches is the potentially adverse effects due to disrupted differentiation of mo-Macs, which are involved in the resolution of inflammation. Blockade of the M-CSF receptor was reported to impair cardiac repair^16^ and skeletal muscle regeneration^17^. Manipulating monocyte fate commitment toward mo-DCs versus mo-Macs would therefore provide an attractive alternative strategy. This would require a better understanding of the molecular regulators orchestrating the monocyte fate decision.

Monocyte fate is not transcriptionally imprinted^18,19^. Instead, monocytes respond to microenvironmental cues that can redirect their fate. Using in vitro models of human monocyte differentiation, we and others have shown that IL-4 signaling is essential to induce mo-DC differentiation^18,20^. Transcription factors involved in this process include IFN regulatory factor 4 (IRF4), aryl hydrocarbon receptor, B lymphocyte-induced maturation protein-1 (BLIMP-1) and the nuclear receptor corepressor 2 (NCOR2)^18,20^. What controls the balance of monocyte differentiation into mo-Macs versus mo-DCs remains unclear.

In the present study, we have identified ETV3 and ETV6 as important regulators of mo-DC differentiation and repressors of monocyte differentiation into macrophages. We provide evidence that ETV3 and ETV6 repress macrophage fate commitment independently of their action on IFN-stimulated gene (ISG) expression. Finally, we validate the role of ETV6 in monocytes for in vivo ISG repression, by analyzing single-cell transcriptomic data from ETV6-mutated patients and in mice deficient for Etv6 in monocytes. We further show that Etv6 in monocytes is required for mo-DC differentiation in a model of inflammatory peritonitis and modulates the severity of symptoms in a model of neuroinflammation. By enabling a better understanding of the molecular ontogeny of monocyte-derived cells, our results should provide opportunities for the therapeutic manipulation of monocyte differentiation.

## Results

### ETV3 and ETV6 expression is greater in mo-DCs than in mo-Macs

**Fig. 1:**
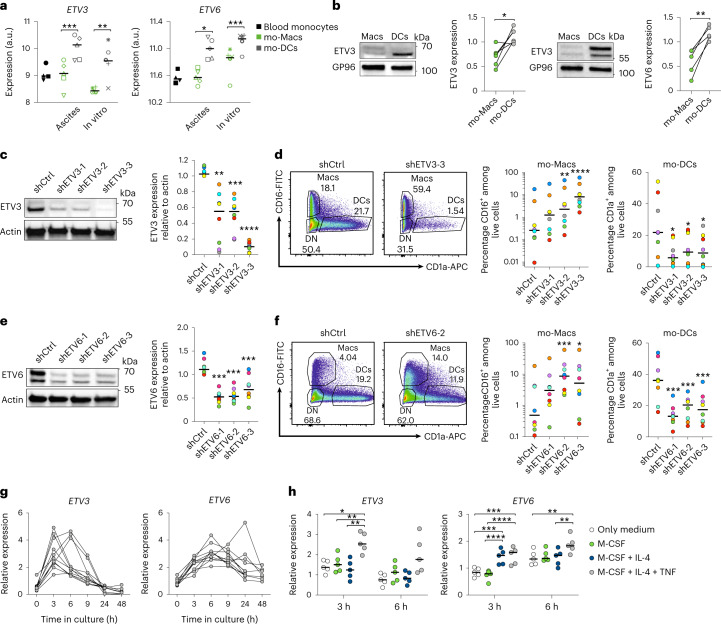
ETV3 and ETV6 are essential for mo-DC differentiation. **a**, Relative expression of ETV3 or ETV6 in blood monocytes, mo-Macs and mo-DCs isolated from peritoneal ascites or generated in vitro (accession nos. GSE102046 and GSE40484). a.u., arbitrary units. **b**–**g**, Monocytes were cultured with M-CSF, IL-4 and TNF. **b**, At day 5, mo-Macs and mo-DCs were sorted and lysed for immunoblot analysis. GP96 was used as a loading control. Representative results are shown (*n* = 5), quantification was performed by densitometry and each symbol represents an individual donor (paired Student’s *t*-test). **c**–**f**, ETV3 or ETV6 expression was silenced using a lentivirus-containing shRNA. **c**,**e**, Protein quantification by immunoblotting after 5 d for ETV3 (c) or ETV6 (e). Actin was used as a loading control. Representative results are shown (*n* = 8), quantification was performed by densitometry and each symbol represents an individual donor (paired Student’s *t*-test). **d**,**f**, Mo-mac and mo-DC differentiation from monocytes after 5 d of ETV3 (d) or ETV6 (f) silencing . One representative donor is shown (*n* = 8) and the median (*n* = 8 in three independent experiments; paired one-way analysis of variance (ANOVA)). DN, double negative. **g**, *ETV3* or *ETV6* mRNA expression was analyzed by RT–qPCR. Each symbol represents an individual donor (*n* = 6 in three independent experiments). **h**, Monocytes were cultured for 3 and 6 h with medium only or combinations of M-CSF, IL-4 and TNF. Each symbol represents an individual donor (*n* = 5 in two independent experiments; paired one-way ANOVA). For all panels: ^*^P < 0.05, ^**^P < 0.01, ^***^P < 0.001, ^****^P < 0.0001. All statistical tests were two sided. For immunoblots, paired samples were derived from the same experiment and processed in parallel. Source data

**Extended Data Fig. 1:**
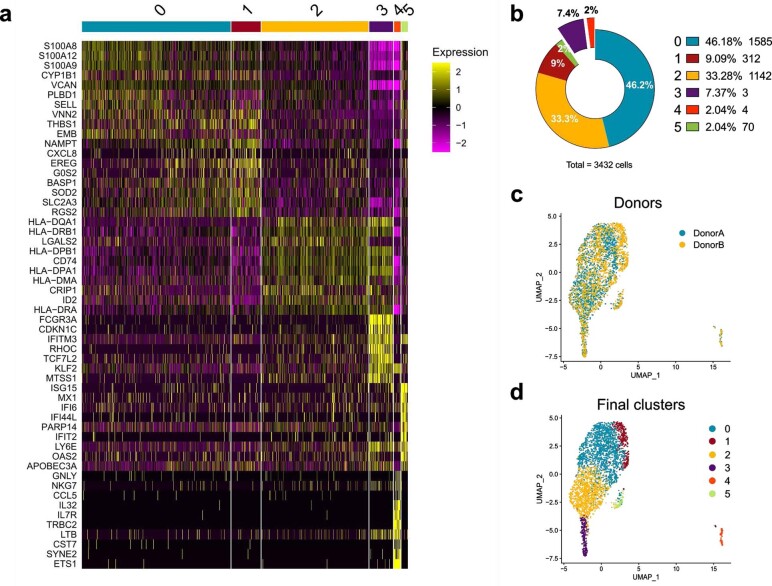
Single-cell RNA-seq analysis of human monocytes. Blood monocytes isolated from 2 individual donors were subjected to single-cell RNA-seq analysis. **(a)** Six clusters were identified. Top differentially expressed between clusters are shown. **(b)** Proportion of each cluster among total cells. **(c)** UMAP representation per donor. **(d)** UMAP representation for the 6 clusters.

We hypothesized that transcription factors differentially expressed between human mo-DCs and mo-Macs could be involved in their differentiation from monocytes. Our transcriptomic analysis of monocyte-derived cells from clinical samples identified *ETV3* and *ETV6* as potential candidates^18^. To assess ETV3 and ETV6 expression, we used our transcriptomics data from cells naturally occurring in vivo in peritoneal ascites^18^. *ETV3* and *ETV6* were more expressed in in vivo mo-DCs compared with mo-Macs (Fig. 1a). To address their potential role in monocyte differentiation, we used our previously published in vitro model allowing the simultaneous differentiation of mo-Macs and mo-DCs^18^. In this model, human CD14^+^ monocytes cultured for 5–6 d with M-CSF, IL-4 and tumor necrosis factor (TNF) differentiate into mo-Macs (CD16^+^) or mo-DCs (CD1a^+^) or remain undifferentiated (double-negative cells). To verify monocyte purity, and in particular the absence of contaminating DC progenitors, we performed single-cell RNA-sequencing (scRNA-seq) on the initial population purified from two different donors (Extended Data Fig. 1a–d). We found two main populations of monocytes displaying high expression of *S100A8* (clusters 0 and 1) or major histocompatibility complex class II (MHC-II) genes (cluster 2; Extended Data Fig. 1a), consistent with the ‘neutrophil-like’ and ‘DC-like’ monocyte populations previously reported^21^. In addition, we identified a small population of *FCGR3A*^+^ monocytes (cluster 3, corresponding to CD14^+^CD16^+^ intermediate monocytes), and a negligable proportion (2% each) of contaminating natural killer (NK) cells (cluster 4) and monocytes with high ISG expression (cluster 5) (Extended Data Fig. 1a,b). These results indicate that our culture model does not contain progenitor cells other than monocytes.

To validate the differential expression of ETV3 and ETV6 in mo-DCs and mo-Macs generated in vitro, we measured their expression in sorted mo-DCs and mo-Macs after differentiation. Both transcription factors were more expressed in mo-DCs compared with mo-Macs at the messenger RNA (Fig. 1a) and protein levels (Fig. 1b).

### ETV3 and ETV6 are essential for human mo-DC differentiation

To address the role of ETV3 or ETV6 in monocyte fate commitment, we silenced their expression using a lentivirus expressing a short hairpin (sh)RNA against ETV3, ETV6 or a scramble sequence. We assessed the effect of silencing on monocyte differentiation after 5 d by staining for phenotypic markers of mo-DCs (CD1a) and mo-Macs (CD16). We used three different shRNAs for each molecule and their efficiency was evaluated by measuring protein expression by immunoblotting (Fig. 1c–f). These shRNAs all significantly decreased ETV3 or ETV6 expression with an efficiency of 40–90% (Fig. 1c,e). Silencing of ETV3 or ETV6 decreased mo-DC and increased mo-Mac differentiation (Fig. 1d,f, respectively). These results show that ETV3 and ETV6 play a key role in mo-DC differentiation. To characterize their expression kinetics during monocyte differentiation, we measured their expression by reverse transcription quantitative PCR (RT–qPCR) at different time points. *ETV3* and *ETV6* mRNA increased during the first hours in culture with a peak at 3 or 6 h for *ETV3* and *ETV6*, respectively (Fig. 1g). To determine which signals increase their expression, we measured *ETV3* and *ETV6* mRNA in monocytes on exposure to M-CSF, in the presence or absence of IL-4 and TNF (Fig. 1h). *ETV3* expression was induced by TNF and *ETV6* expression by IL-4, with TNF sustaining its expression at later time points. These results show that ETV3 and ETV6 are expressed on exposure to inflammatory signals at an early stage of monocyte differentiation, suggesting that they could play a role in their lineage commitment toward mo-DCs.

### ETV3 and ETV6 repress ISGs

**Extended Data Fig. 2:**
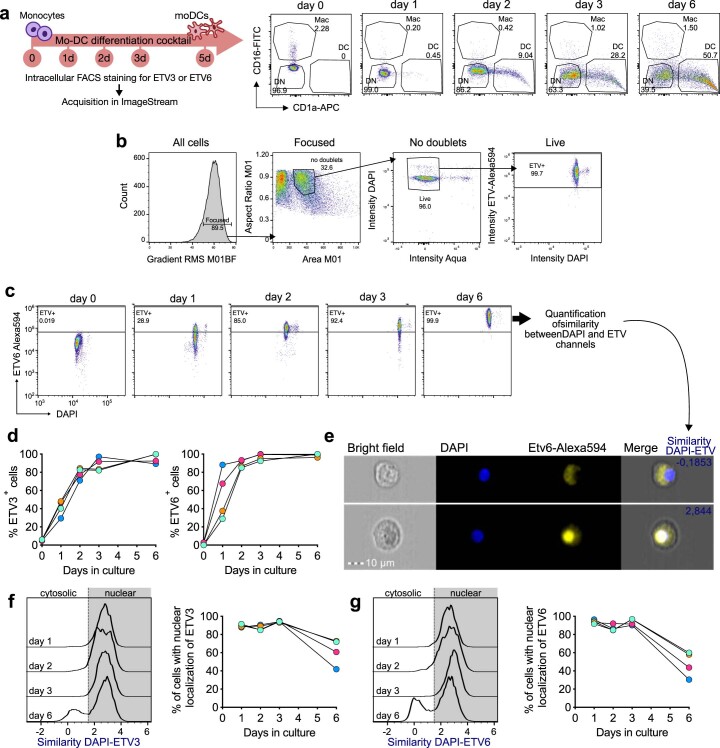
ETV3 and ETV6 exert their function at an early stage of monocyte differentiation. Monocytes were cultured for 6 days with a modified cocktail favoring mo-DC differentiation (M-CSF, IL-4 and increased concentration of TNFα). **(a)** Experimental set up. Monocyte differentiation at the indicated time points. One representative donor is shown (n = 4 in 2 independent experiments). **(b)** Gating strategy for imaging flow cytometry. One representative donor is shown (n = 4 in 2 independent experiments). **(c)** Kinetics of ETV6 expression. One representative donor is shown (n = 4 in 2 independent experiments). **(d)** Percentage of ETV3+ or ETV6+ cells across time. Each dot represents an individual donor (n = 4 in 2 independent experiments). **(e)** Representative images of cells displaying cytosolic or nuclear localization of ETV6 at day 6 (n = 4 in 2 independent experiments). Similarity of DAPI and ETV6 stainings is indicated for each cell. **(f-g)** Representative histograms showing the similarity of DAPI and ETV3 stainings (f) or of DAPI and ETV6 stainings (g). Percentage of cells with nuclear localization is shown. Each dot represents an individual donor (n = 4 in 2 independent experiments).

To decipher the transcriptional network of ETV3 and ETV6, we first investigated the kinetics of their nuclear localization using imaging flow cytometry. To increase the resolution of our analysis, we sought to favor mo-DC differentiation in the culture system by using a modified cytokine cocktail (increased TNF concentration) (Extended Data Fig. 2a). We performed intracellular staining of ETV3 or ETV6 after 0, 1, 2, 3 or 6 d of culture. To quantify the expression of ETV3 and ETV6, we gated on ETV3- or ETV6-positive cells (Extended Data Fig. 2b,c). The percentage of ETV3^+^ and ETV6^+^ cells increased gradually, reaching a plateau at day 3 (Extended Data Fig. 2d). To quantify the nuclear localization of ETV3 or ETV6, we used the ImageStream software to calculate the ‘similarity’ of the ETV3 or ETV6 channel with the nuclear DAPI staining. High similarity (>1.8) indicates a nuclear localization of the transcription factor, whereas low similarity (<1.8) indicates a cytosolic localization (Extended Data Fig. 2e). We observed that ETV3 and ETV6 are located in the nucleus until day 3 in around 90% of the cells (Extended Data Fig. 2f,g). By contrast, at day 6, ETV3 and ETV6 are located in the cytosol in around 50% of the cells. As the transcriptional activity of ETV3 and ETV6 requires their nuclear localization, this observation suggests that ETV3 and ETV6 exert their function mainly during the first days of differentiation.

**Fig. 2:**
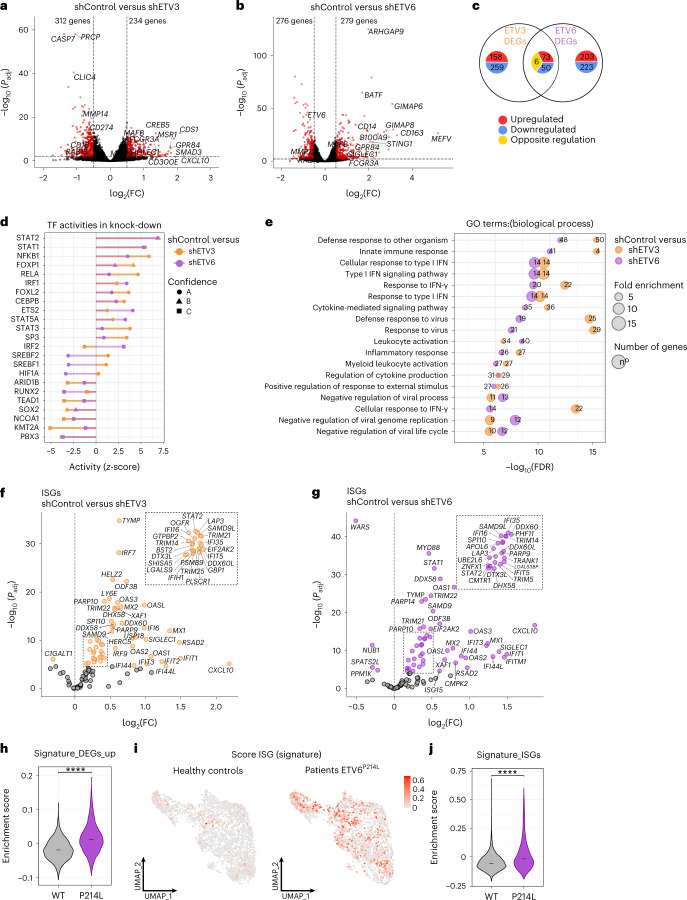
ETV3 and ETV6 repress ISG expression in human monocytes. **a**–**g**, Monocytes cultured with M-CSF, IL-4 and TNF for 3 d. ETV3 or ETV6 expression was silenced using a lentivirus-containing shRNA. Bulk RNA-seq analysis was performed using five individual donors. **a**,**b**, Volcano plot showing DEGs obtained with DESeq2 (Wald’s test) between shControl and shETV3 (a) or shETV6 (b). **c**, Overlap of DEGs on ETV3 or ETV6 silencing. Up- or downregulation was calculated compared with the control condition. **d**, Inference of transcription factor activity with DoRoThEA. Activity (*z*-score) in silenced samples compared with the control is shown for top regulons (ETV3 in orange, ETV6 in purple). A, highest confidence; C, lowest confidence. **e**, Enrichment of the top GO terms (biological process) associated with genes upregulated in silenced compared with control samples. Fold enrichment is indicated by the size of the circle. The number of genes observed is indicated for each pathway. FDR, false discovery rate. **f**,**g**, Volcano plot of ISGs among DEGs for ETV3 (**f**) or ETV6 (**g**) silencing, obtained with DESeq2 (Wald’s test). Colored dots indicate *P*_adj_ < 0.05. **h**–**j**, ScRNA-seq data from blood monocytes of healthy (gray) and mutant ETV6^P214L^ (purple) patients downloaded from a public source. **h**, Enrichment score for the list of upregulated genes on ETV6 silencing in human monocyte culture. The median is shown. **i**,**j**, Enrichment score for the ISG signature in individuel cells (i) andmedian (j) (Student’s *t*-test: ^****^P < 0.0001). All statistical tests were two sided.

**Extended Data Fig. 3:**
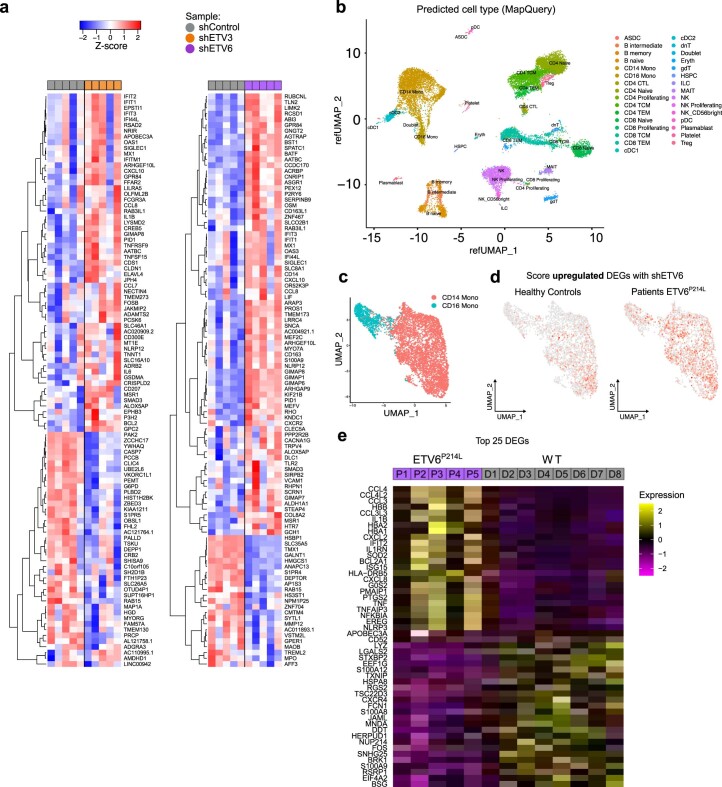
Transcriptomic analysis of ETV3 or ETV6-deficient human monocytes. **(a)** Top differentially expressed genes between silenced conditions and ETV3 or ETV6 silencing (n = 5). **(b-e)** Analysis of public scRNAseq data of PBMCs from healthy and Etv6^P214L^ patients. **(b)** Reference UMAP visualization of healthy and ETV6^P214L^ PBMCs with annotated clusters. **(c)** Subsetting and remapping of CD14+ and CD16+ monocytes. **(d)** Expression feature plot of the upregulated genes upon ETV6 silencing in monocyte culture. **(e)** Top differentially expressed genes between healthy (WT) and ETV6^P214L^ monocytes (n = 8 for healthy and n = 5 for ETV6^P214L^ mutation).

To identify the target genes of *ETV3* and *ETV6*, we performed transcriptomic analysis by bulk RNA-seq on monocytes silenced or not silenced for ETV3 or ETV6, at day 3 of differentiation, with the modified cytokine cocktail to favor mo-DC development. Then, we performed a differential gene expression analysis using DESeq2 comparing control with silenced samples for ETV3 (Fig. 2a) or ETV6 (Fig. 2b) separately. We defined the differentially expressed genes (DEGs) by a log_2_(fold-change) (log_2_(FC)) > 0.5 and a *P*_adj_ < 0.05. Comparison of the DEGs for each shRNA revealed unique transcriptional networks, because most of the genes are specific for ETV3 or ETV6 silencing (Fig. 2c and Extended Data Fig. 3a; lists of DEGs can be found in Supplementary Tables 1 and 2).

To identify the molecular pathways controlled by ETV3 or ETV6, we performed network analysis. We calculated transcription factor activity using DoRoThEa regulons and VIPER^22^ (Fig. 2d). STAT1 and STAT2 were the most active transcription factors in silenced samples. We then calculated the enrichment of gene ontology (GO) terms (biological process) for upregulated genes (Fig. 2e). Type I IFN response gene sets were enriched in silenced samples. This is consistent with the predicted activity of STAT1 and STAT2, which are known to control the expression of ISGs^23^. To confirm this, we filtered the DEG matrix for known ISGs. Most of the ISGs were expressed more in silenced compared with control samples (Fig. 2f,g). To determine the in vivo relevance of this finding, we reanalyzed scRNA-seq data from peripheral blood mononuclear cells (PBMCs) of patients carrying a germline mutation of *ETV6* (P214L) resulting in loss of function^24^. We first filtered the data to retain only CD14^+^ and CD16^+^ monocytes from healthy and ETV6^P214L^ patients (Extended Data Fig. 3b,c). We then interrogated the single-cell data of ETV6^P214L^ and wild-type (WT) monocytes with different gene sets. Genes upregulated on ETV6 silencing in our in vitro system were enriched in ETV6^P214L^ monocytes compared with WT monocytes (Fig. 2h and Extended Data Fig. 3d). Moreover, ETV6^P214L^ monocytes had a higher enrichment for ISGs than WT monocytes (Fig. 2i,j and Extended Data Fig. 3e), consistent with a previous report^24^. These results show that ETV3 and ETV6 repress ISG expression in monocytes in vitro and in vivo in humans and suggest that STAT1 signaling may be involved in the differentiation of monocytes.

### The type I IFN pathway promotes mo-Mac differentiation

**Fig. 3:**
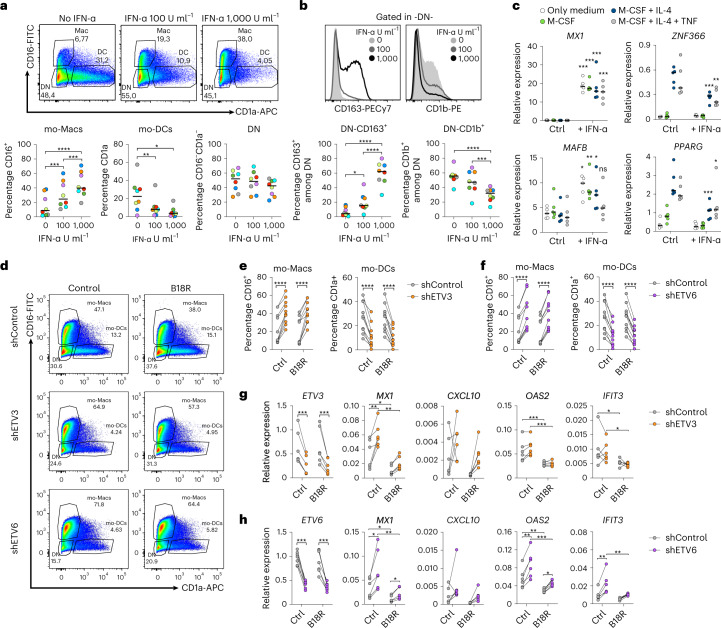
ETV3 and ETV6 control monocyte differentiation independently of their effect on ISGs. **a**,**b**, Monocytes cultured for 5 d with M-CSF, IL-4 and TNF, in the absence or presence of 100 or 1,000 U ml^−1^ of IFN-α. **a**, Monocyte differentiation. DN, double negative. One representative donor is shown (*n* = 8). Percentage of mo-Mac, mo-DC or DN cells at day 5. The median is shown (*n* = 8 in three independent experiments; paired one-way ANOVA). **b**, Expression of CD163 and CD1b in the DN cells. Histograms of one representative donor are shown (*n* = 8). Percentage of CD163^+^ or CD1b^+^ cells among DN cells. The median is shown (*n* = 8 in three independent experiments; paired one-way ANOVA). **c**, Monocytes were cultured with medium only or combinations of M-CSF, IL-4 and TNF, in the presence or absence of 1,000 U ml^−1^ of IFN-α. *MX1* and *MAFB* expression is measured by RT–qPCR after 3 h and *PPARG* and *ZNF366* after 6 h. The median is shown (*n* = 5 in two independent experiments; paired two-way ANOVA). The asterisks represent tests against the same condition without IFN-α. Ctrl, Control. **d**–**h**, Monocytes cultured with M-CSF, IL-4 and TNF for 5 d, in the presence or absence of recombinant B18R. ETV3 or ETV6 expression was silenced using a lentivirus-containing shRNA. **d**, Monocyte differentiation. One representative donor is shown (*n* = 9). **e**,**f**, Percentage of mo-Macs, mo-DCs or DN cells at day 5 after ETV3 (e) or ETV6 (f) silencing. The median is shown (*n* = 9 in three independent experiments; paired Student’s *t*-test). **g**,**h**, Expression of *ETV3*, *ETV6* and selected ISGs measured by RT–qPCR after ETV3 (g) or ETV6 (h) silencing (*n* = 5 for ETV3 and 6 for ETV6; paired two-way ANOVA). For all panels: ^*^P < 0.05, ^**^P < 0.01, ^***^P < 0.001, ^****^P < 0.0001. All statistical tests were two sided.

**Extended Data Fig. 4:**
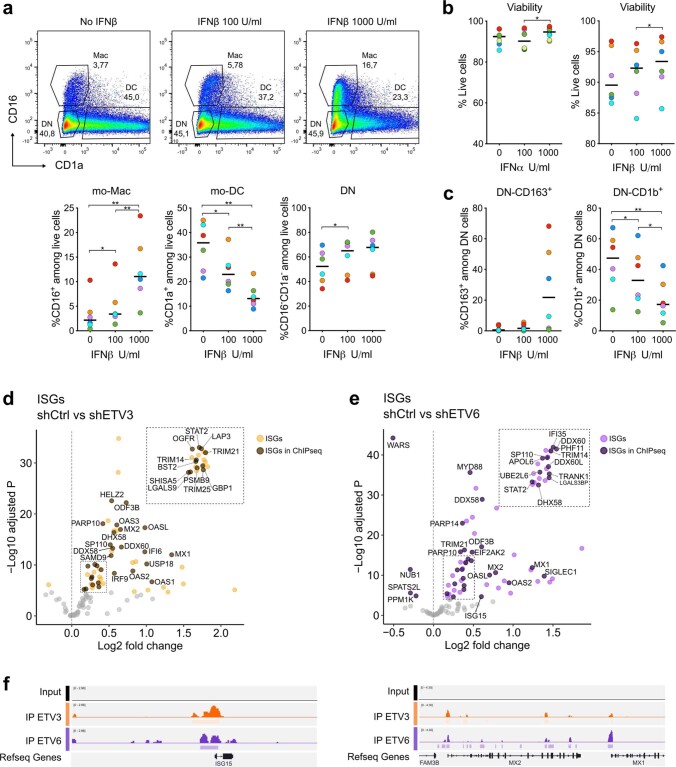
Type I interferon increases mo-Mac differentiation and ETV3 and ETV6 repress interferon-stimulated genes. **(a-c)** Monocytes were cultured for 5 days with M-CSF, IL-4 and TNFα, in absence or presence of 100 or 1000 U/mL IFNα or IFNβ. **(a)** Monocyte differentiation after 5 days. DN = double negative. One representative donor is shown (n = 6). Percentage of mo-Mac, mo-DC or DN cells. Median is shown (n = 6 in 2 independent experiments). Paired-one way Anova. **(b)** Percentage of live cells. Each symbol represents an individual donor (n = 6). Median is shown. Paired-one way Anova. **(c)** Percentage of CD163+ or CD1b+ cells among DN cells. Median is shown (n = 6 in 2 independent experiments). Paired-one way Anova. **(d-f)** Monocytes were cultured with M-CSF, IL-4 and TNFα for 3 days. ChIP-seq analysis was performed for ETV3 or ETV6. **(d-e)** Overlap of differentially expressed ISG found in RNA-seq and genes identified in ChIP-seq for ETV3 (d) and ETV6 (e) (Wald test). **(f)** Gene tracks from ChIP-seq analysis for the genomic regions of ISG15, MX1 and MX2. IP = immuno-precipitation. All statistical tests were two-sided.

Given the impact of ETV3 or ETV6 silencing on mo-DC differentiation, our findings suggest that the type I IFN pathway may be activated in our model despite the absence of exogenous IFN in the culture system. However, we could not detect type I IFN secretion in the culture supernatant (not shown). To directly assess the effect of STAT1 activation on monocyte differentiation, we cultured monocytes in the presence of IFN-α or IFN-β. Type I IFN increased mo-Mac and decreased mo-DC differentiation in a dose-dependent manner (Fig. 3a and Extended Data Fig. 4a). Neither IFN-α nor IFN-β affected monocyte-derived cell viability (Extended Data Fig. 4b). In addition, type I IFN increased the expression of CD163, an early mo-Mac marker, and decreased the expression of CD1b, an early mo-DC marker, on the double-negative cells (Fig. 3b and Extended Data Fig. 4c). Collectively, these results show that activation of the type I IFN pathway promotes mo-Mac differentiation at the expense of mo-DCs. To understand how STAT1 signaling could affect monocyte fate decision, we exposed monocytes to IFN-α, in the presence or absence of cytokines, and measured the early expression of *MAFB* and IL-4-induced genes *PPARG* and *ZNF366* (Fig. 3c). *MAFB* was rapidly increased on IFN-α exposure, along with the ISG *MX1*, whereas *PPARG* and *ZNF366* expression on IL-4 treatment was inhibited by IFN-α. These results suggest that STAT1 activation modulates the mo-Mac:mo-DC balance by repressing the IL-4-induced mo-DC differentiation program.

### Monocyte differentiation is controlled independently of ISGs

To directly test whether ISG expression plays a role in the control of monocyte differentiation by ETV3 or ETV6, we sought to inhibit type I IFN signaling in our culture model using recombinant viral B18R, a soluble receptor of type I IFN that prevents signaling through the IFN-α/β receptor (IFNAR)^25^. Exposure to B18R did not impact the proportions of mo-DCs and mo-Macs obtained with or without silencing of ETV3 or ETV6 (Fig. 3d–f), even though B18R efficiently inhibited ISG expression, including *MX1, CXCL10, OAS2* and *IFIT3* (Fig. 3g,h). These results indicate that inhibition of the type I IFN pathway does not rescue mo-DC differentiation in the absence of ETV3 or ETV6 expression. We conclude that ETV3 and ETV6 regulate monocyte differentiation independently of their action on ISGs.

### ETV3 and ETV6 repress mo-Mac program and differentiation

**Fig. 4:**
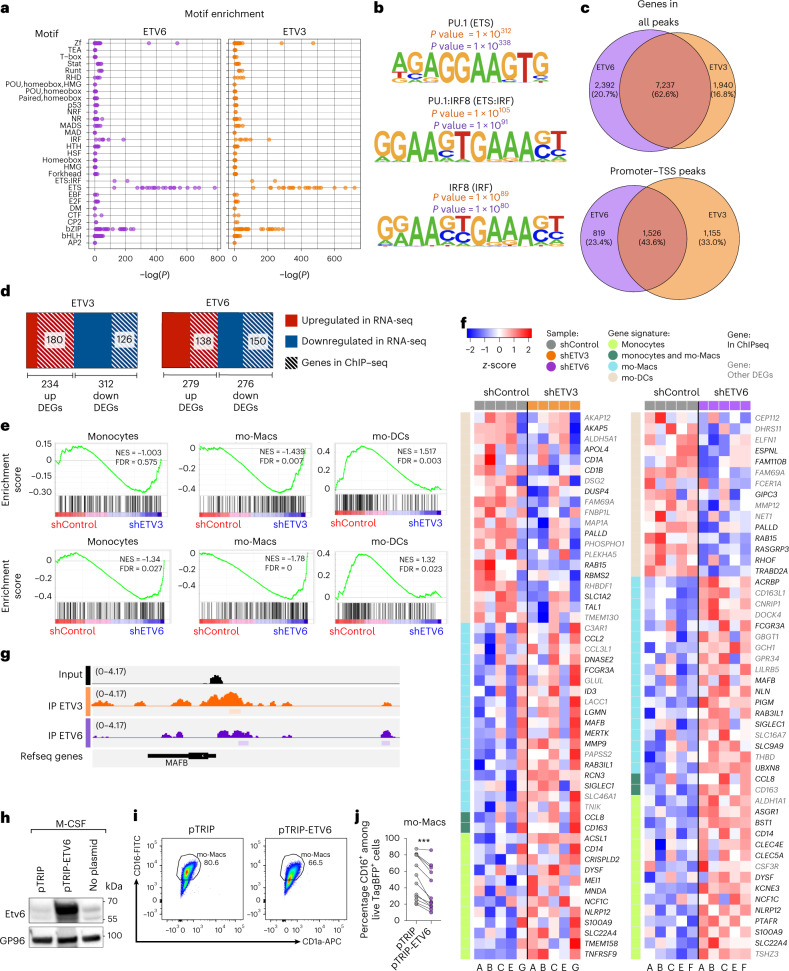
ETV3 and ETV6 repress mo-Mac transcriptional program and differentiation. **a**–**d**,**f**,**g**, Monocytes were cultured with M-CSF, IL-4 and TNF for 3 d. ChIP–seq analysis was performed for ETV3 or ETV6. **a**, Motif enrichment analysis obtained with HOMER. **b**, Most enriched motifs. **c**, Overlap of identified genes for ETV3 or ETV6 immunoprecipitation (IP). Peaks in all gene regions or only in the transcription start site (TSS) region are shown. **d**, Overlap of DEGs found in RNA-seq and genes identified in ChIP–seq. **e**,**f**, Data from RNA-seq analysis. **e**, GSEA of monocyte, mo-Mac and mo-DC signatures in control (red) versus silenced (blue) samples. NES, normalized enrichment score. **f**, Heatmap of DEGs belonging to the monocyte, mo-Mac or mo-DC signatures. Samples were ordered by condition and genes were ordered manually. Genes detected in ChIP–seq analysis are emboldened. **g**, Gene tracks from ChIP–seq analysis for the genomic region of *MAFB*. **h**–**j**, Monocytes were cultured with M-CSF for 5 d. ETV6 was overexpressed using a lentivirus containing an expression plasmid. **h**, Protein quantification by immunblotting. GP96 was used as a loading control. Representative results are shown (*n* = 5 in two independent experiments). Paired samples derive from the same experiment and were processed in parallel. **i**, Mo-Mac differentiation. One representative donor is shown (*n* = 11). **j**, Percentage of mo-Macs after 5 d (*n* = 11 in four independent experiments; paired Student’s *t*-test: ^***^P < 0.001). All statistical tests were two sided. Source data

To identify the genes directly targeted by ETV3 and ETV6 during monocyte differentiation, we performed chromatin immunoprecipitation followed by sequencing (ChIP–seq) at day 3 of differentiation with the modified cytokine cocktail favoring mo-DC differentiation (a complete list of peaks can be found in Supplementary Table 3). We first analyzed the enrichment in known motifs using HOMER (Fig. 4a,b). We found that the ETS motif was the most enriched in both ETV3 and ETV6 ChIP–seq datasets, with the ETS–IRF composite motif and IRF8 motif also significantly enriched, consistent with a previous report of interaction between ETV6 and IRF8 in macrophages^26^. Most identified genes were common between ETV3 and ETV6 (Fig. 4c), including at promoter regions. To assess the targets of ETV3 and ETV6 among DEGs, we intersected a list of genes from the RNA-seq and ChIP–seq datasets (Fig. 4d). We found that around 50% of regulated genes were directly bound by ETV3 or ETV6. Consistent with the transcriptomics analysis, ISGs were found among direct targets of ETV3 and ETV6 (Extended Data Fig. 4d–f).

To evaluate how ETV3 and ETV6 modulate monocyte fate commitment, we performed gene set enrichment analysis (GSEA) on DEGs and assessed the enrichment of monocyte, mo-Mac or mo-DC gene signatures. The mo-Mac signature was enriched in silenced samples, whereas mo-DC genes were enriched in the control condition (Fig. 4e). Among DEGs belonging to these signatures, we found a vast majority of direct targets identified in ChIP–seq (Fig. 4f). In particular, we found that *MAFB* expression, which is essential for mo-Mac differentiation^18^, was directly modulated by ETV3 and ETV6 (Fig. 4f,g). These results suggest that ETV3 and ETV6 repress the mo-Mac transcriptional program. To confirm this, we sought to overexpress ETV6 during monocyte differentiation. To avoid spontaneous expression of ETV6, we cultured monocytes with M-CSF only, a condition in which monocytes differentiate exclusively into mo-Macs. We validated the forced expression of ETV6 by immunoblotting (Fig. 4h). ETV6 overexpression decreased mo-Mac differentiation (Fig. 4i,j). Taken together, these results indicate that ETV3 and ETV6 directly repress mo-Mac differentiation.

### Etv6 represses ISG expression in mice

**Fig. 5:**
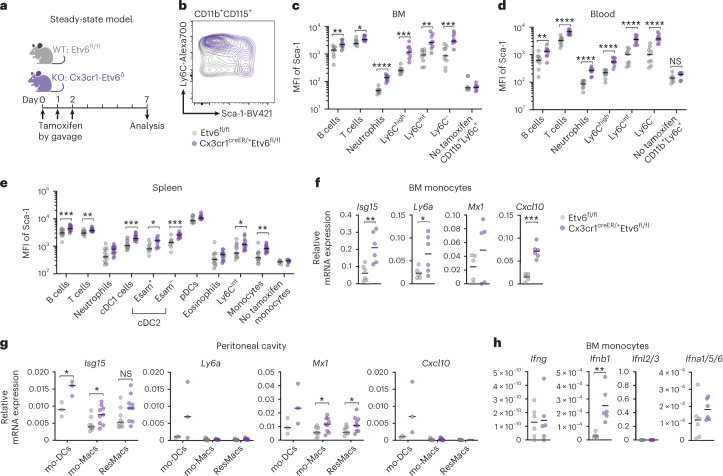
Etv6 represses ISG expression in mice. **a**, Experimental set-up. Etv6^fl/fl^ (WT) and Cx3cr1-Etv6^Δ^ (KO) mice gavaged with tamoxifen on 3 consecutive days and analyzed on day 7. **b**–**e**, Sca-1 expression in immune cells from WT (gray) and KO (purple) mice. **b**, Sca-1 expression in BM monocytes from WT and KO mice. Results from one representative pair of littermates are shown (*n* = 16). **c**–**e**, Mean fluorescence intensity (MFI) of Sca-1 shown for immune cell types in BM (**c**), blood (**d**) and spleen (**e**). Each symbol represents one mouse (*n* = 16 in four independent experiments, except for the ‘no tamoxifen’ condition, *n* = 11 in three independent experiments; unpaired Student’s *t*-test between WT and KO). **f**,**g**, Expression of selected ISGs in BM monocytes (**f**) or peritoneal mo-DCs, mo-Macs and resident macrophages (ResMac) (**g**) measured by RT–qPCR. Each symbol represents one mouse (**f**: *n* = 6 in two independent experiments; **g**: *n* = 3–9 in three independent experiments). The median is shown; unpaired Student’s *t*-test between WT and KO. **h**, Expression of IFN genes in BM monocytes. The median is shown; unpaired Student’s *t*-test between WT and KO. For all panels: ^*^P < 0.05, ^**^P < 0.01, ^***^P < 0.001, ^****^P < 0.0001. All statistical tests were two sided. NS, not significant.

**Extended Data Fig. 5:**
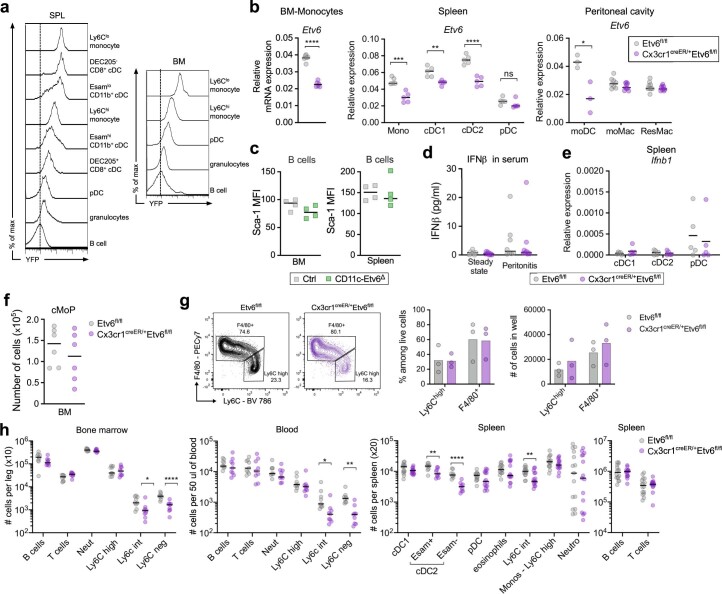
Characterization of Cx3cr1-Etv6^Δ^ mice. **(a)** YFP expression in untreated Cx3cr1-CreER mice. Representative staining profiles of YFP expression in spleen (SPL) and bone marrow (BM). **(b)** Expression of Etv6 measured by RT-qPCR in bone marrow monocytes (n = 6 in 3 independent experiments), spleen monocytes and DC subsets (n = 5 in 2 independent experiments) and peritoneal cavity (n = 8 in 3 independent experiments, except for mo-DC n = 3 as cells were pooled in 3 independent experiments). **(c)** Mean Fluorescence Intensity (MFI) of Sca1 expression in mature B cells (B220^+^MHCII^high^) in Etv6^fl/fl^ (Ctrl) and CD11c^−^Cre^+/−^ Etv6^fl/−^ (CD11c^−^Etv6^Δ/−^) spleen and bone marrow (median is shown, n = 4). **(d)** IFNβ quantification in serum of WT and Cx3cr1-Etv6^Δ^ mice at steady state or during inflammation (thioglycolate-induced peritonitis). **(e)** Expression of *Ifnb1* measured by RT-qPCR in spleen DC subsets (n = 5 in 2 independent experiments). **(f)** Numbers of monocyte progenitors (cMoP) in the bone marrow of WT and Cx3cr1-Etv6^Δ^ mice (median is shown, n = 6). **(g)** cMoP were isolated from the bone marrow of WT and Cx3cr1-Etv6^Δ^ mice and cultured for 3 days in the presence of M-CSF. Differentiation was assessed by flow cytometry. Percentage and number of recovered cells are shown (3 independent experiments). **(h)** Etv6^fl/fl^ (WT) and Cx3cr1-Etv6^Δ^ (KO) mice were gavaged with tamoxifen on 3 consecutive days and analyzed on day 7. Immune cell counts in WT (grey) and KO (purple) mice in bone marrow, blood and spleen (Neutro = neutrophils). Each symbol represents one mouse (n = 11-16 in at least 3 independent experiments). Median is shown. Unpaired t-test between WT and KO. All statistical tests were two-sided.

**Extended Data Fig. 6:**
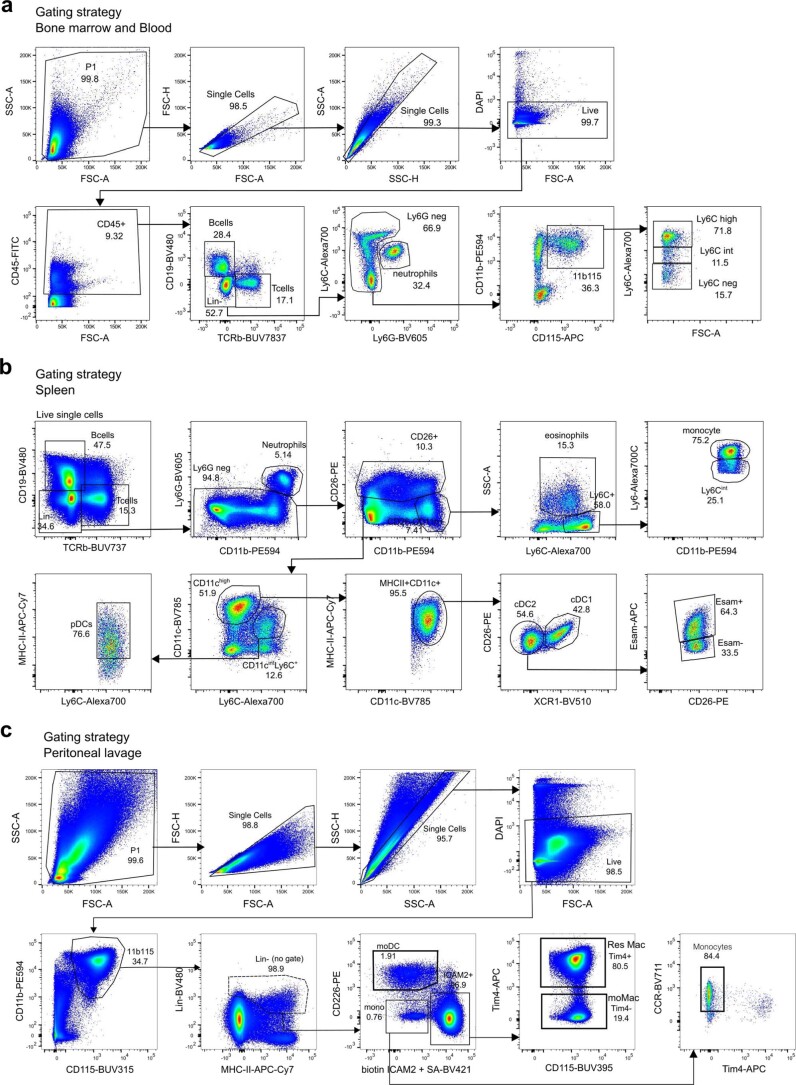
Gating strategies of immune cells in mouse bone marrow, blood, spleen and peritoneal lavage. **(a)** Gating strategy for bone marrow and blood cell suspensions. Cells were separated into B and T cells, neutrophils, and Ly6C high, intermediate (int) and negative (neg) monocytes. **(b)** Gating strategy for spleen cell suspensions. Doublets and dead cells were excluded as in (a). Cells were separated into B and T cells, neutrophils, eosinophils, Ly6C^high^ and Ly6C^int^ monocytes, pDC, cDC1 and Esam^+^ or Esam^−^ cDC2. **(c)** Gating strategy for peritoneal lavage analysis. Lineage positive cells (expressing TCRβ, CD19, NK1.1 and Siglec-F) were excluded from CD11b+ CD115+ cells. Cell were separated into monocytes (Mono CCR2^+^), mo-DC, mo-Mac (ICAM2^+^Tim4^−^) and resident macrophages (Res Mac, ICAM2^+^TIM4^+^).

To validate the physiological relevance of our findings, we employed a mouse model that deletes Etv6 in Cx3cr1-expressing cells after induction with tamoxifen (Fig. 5a). Cx3cr1 is a canonical marker of patrolling monocytes, thus the deletion of Etv6 in Cx3cr1^+^ cells would be expected to delete Etv6 in monocytes and their progeny^27^. To confirm the cell types targeted by the deletion, we measured a yellow fluorescent protein (YFP) reporter mimicking the endogenous Cx3cr1 expression pattern. As expected, YFP was expressed at the highest level in Ly6c^low^ patrolling monocytes, as well as in certain DC subsets including Esam^−^ splenic cDC2 cells (Extended Data Fig. 5a). In addition, we measured *Etv6* expression by RT–qPCR in cell-sorted populations (Extended Data Fig. 5b). *Etv6* expression was significantly decreased in bone marrow (BM) and spleen monocytes of Cx3cr1-Etv6^Δ^ mice, as well as in spleen cDC1 and cDC2 cells but not pDCs. We have previously identified a population of peritoneal mo-DCs^18^. *Etv6* was also significantly decreased in peritoneal mo-DCs of Cx3cr1-Etv6^Δ^ mice but not in peritoneal mo-Macs or resident macrophages (Extended Data Fig. 5b). To assess the impact of Etv6 deletion in Cx3cr1-expressing cells on ISG expression in vivo, we measured by flow cytometry the expression of Sca-1, an IFN-inducible protein^28,29^. We analyzed immune cells from WT and Cx3cr1-Etv6^Δ^ BM, blood and spleen (gating strategies in Extended Data Fig. 6a–c). Sca-1 expression was higher in Cx3cr1-Etv6^Δ^ than in WT BM monocytes (Fig. 5b). Sca-1 was also more expressed in Cx3cr1-Etv6^Δ^ mice in B cells, T cells and neutrophils in BM, blood and spleen, and in spleen cDC1 and cDC2 cells and pDCs (Fig. 5c–e). By contrast, deletion of Etv6 in CD11c-expressing cells did not affect Sca-1 expression in BM and spleen B cells (Extended Data Fig. 5c). These results indicate that the increased ISG expression in Cx3cr1-Etv6^Δ^ mice is due to Etv6 deletion in monocytes rather than in DCs. To confirm our observations, we analyzed the expression of additional ISGs by RT–qPCR in BM monocytes and in peritoneal mo-DCs, mo-Macs and resident macrophages. *Isg15*, *Mx1*, *Cxcl10* and *Ly6a* (encoding Sca-1) were expressed more in Etv6^Δ^ than in WT monocytes (Fig. 5f) and in peritoneal Etv6^Δ^ mo-DCs compared with WT cells (Fig. 5g). *Isg15* and *Mx1* were also expressed more in Etv6^Δ^ peritoneal macrophages (Fig. 5g). This widespread spontaneous ISG expression suggests that Etv6 deletion induces type I IFN secretion by Cx3cr1^+^ cells. We were unable to detect circulating IFN-β (Extended Data Fig. 5d); however, we found that *Ifnb1* spontaneously expressed in BM Etv6^Δ^ monocytes (Fig. 5h), but not in spleen DCs (Extended Data Fig. 5e). Collectively, these results show that Etv6 represses type I IFN responses in vivo in the steady state.

### Etv6 controls mo-DC differentiation in mice

**Fig. 6:**
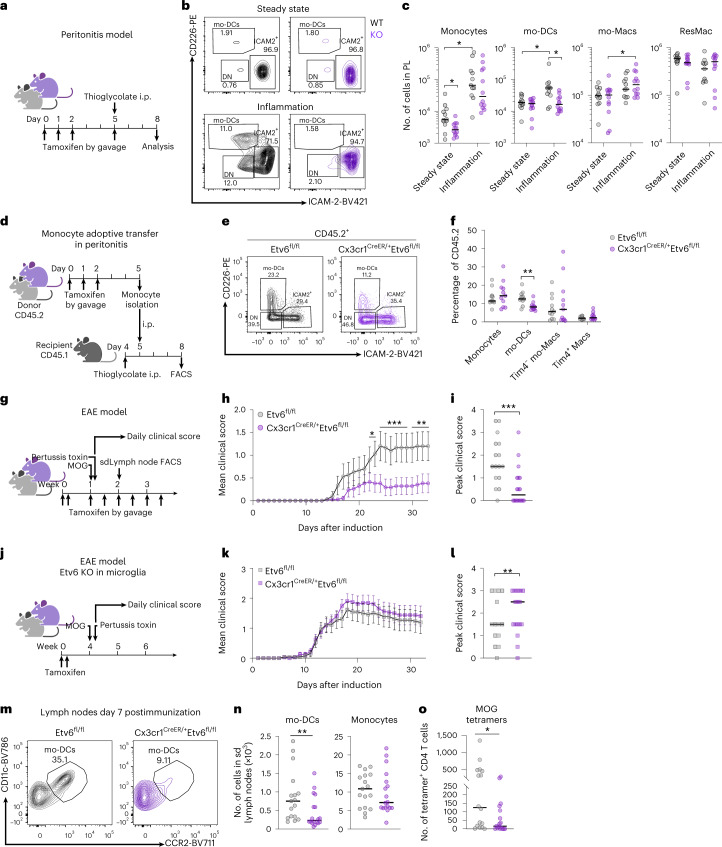
Etv6 controls mo-DC differentiation during inflammation in mice. **a**, Experimental set-up of peritonitis model. i.p., intraperitoneally. **b**, CD226 and ICAM-2 expression in CD11b^+^CD115^+^ cells from peritoneal lavage of Etv6^fl/fl^ (WT) and Cx3cr1-Etv6^Δ^ (KO) mice. The results from one representative pair of littermates are shown for each setting. **c**, Numbers of monocytes, mo-DCs, mo-Macs or resident macrophages (ResMac) in the peritoneal lavage. Each symbol represents one mouse. The median is shown (*n* = 12 in three independent experiments; unpaired Student’s *t*-test between WT and KO; two-way ANOVA with Tukey’s posttest between steady-state and inflammation groups). **d**–**f**, Monocytes purified from the BM of WT or KO mice adoptively transferred into the inflamed peritoneum of recipient mice. **d**, Experimental set-up. Transferred cells were distinguished using congenic markers CD45.1 and CD45.2. **e**, CD226 and ICAM-2 expression in CD45.2^+^CD11b^+^CD115^+^ cells from peritoneal lavage of recipient mice. The results from one representative mouse are shown. **f**, Percentage of monocytes, mo-DCs, Tim4^−^ or Tim4^+^ macrophages among CD45.2^+^ cells in the peritoneal lavage of recipient mice. Each symbol represents one mouse. The median is shown (*n* = 11 in three independent experiments; unpaired Student’s *t*-test between WT and KO). **g**–**o**, EAE was induced by injection of MOG peptide. **g**, Experimental set-up for Etv6 deletion in all target cells. **h**, The mean clinical score is shown and bars represent the s.e.m. sd, site-draining (*n* = 16 for WT and 18 for KO in four independent experiments; multiple Mann–Whitney *U*-tests between WT and KO groups). **i**, Peak clinical score. The median is shown. Each dot represents one mouse (median of *n* = 16 for WT and *n* = 18 for KO in four independent experiments; Mann–Whitney *U*-test). **j**, Experimental set-up for Etv6 deletion in microglia. **k**, The mean clinical score is shown and the bars represent the s.e.m. (*n* = 14–15 in two independent experiments). **l**, Peak clinical score. The median is shown and each dot represents one mouse (*n* = 14–15 in two independent experiments; Mann–Whitney *U*-test). **m**–**o**, Lymph nodes draining the site of the MOG injection analyzed 7 d after immunization. **m**, CD11c and CCR2 expression in CD11c^+^MHC-II^−^CD26^−^ cells. Results are from one representative pair of littermates. **n**, Numbers of mo-DCs and monocytes in lymph nodes (*n* = 17–18 in three independent experiments; Mann–Whitney *U*-test). **o**, Number of MOG-tetramer^+^CD4^+^ T cells in lymph nodes (*n* = 17–18 in three independent experiments; Mann–Whitney *U*-test). For all panels: ^*^P < 0.05, ^**^P < 0.01, ^***^P < 0.001, ^****^P < 0.0001. All statistical tests were two sided.

To determine whether Etv6 modulates monocyte differentiation in vivo, we first analyzed monocyte populations in steady-state blood, bone marrow (BM) and spleen of Cx3cr1-Etv6^Δ^ mice. The number of monocyte progenitors (cMoPs) in the BM was unchanged (Extended Data Fig. 5f). The differentiation of monocytes from the cMoPs of Cx3cr1-Etv6^Δ^ or WT mice was also similar in an in vitro assay (Extended Data Fig. 5g), excluding a role for Etv6 in the differentiation of cMoPs into monocytes. The numbers of B cells, T cells, neutrophils or Ly6C^high^ monocytes were not affected by Etv6 deletion (Extended Data Fig. 5h). The numbers of CD11b^+^CD115^+^Ly6C^int^ and CD11b^+^CD115^+^Ly6C^neg^ monocytes decreased in Cx3cr1-Etv6^Δ^ mice compared with WT mice. Moreover, the spleens of Cx3cr1-Etv6^Δ^ mice harbored decreased numbers of cDC2 cells, particularly of the Esam^−^ subset that is transcriptionally and functionally related to monocytes (Extended Data Fig. 5h)^30^. To address the role of Etv6 in monocyte differentiation in vivo, we analyzed the peritoneal compartment in the steady state and during inflammation (Fig. 6a). In Cx3cr1-Etv6^Δ^ mice, mo-DCs and mo-Macs in the steady-state peritoneum were unaffected (Fig. 6b). By contrast, during thioglycolate-induced peritonitis, numbers of mo-DCs increased only in WT mice, whereas mo-Macs increased in Cx3cr1-Etv6^Δ^ mice (Fig. 6c). Monocyte recruitment to the inflamed peritoneum did not differ between WT and Cx3cr1-Etv6^Δ^ mice (Fig. 6c), suggesting that the mo-DC:mo-Mac balance was skewed in Cx3cr1-Etv6^Δ^ mice. To confirm that this phenomenon was a monocyte-intrinsic effect, we performed adoptive transfer of CD45.2^+^ WT or Etv6^Δ^ monocytes into the inflamed peritoneum of CD45.1^+^ recipient mice (Fig. 6d). Transferred monocytes differentiated in situ into mo-DCs and mo-Macs (Fig. 6e); however, the mo-DC output was significantly decreased in the progeny of Etv6^Δ^ monocytes compared with WT ones (Fig. 6f). These results show that Etv6^Δ^ monocytes are impaired in their differentiation into mo-DCs during inflammation in mice, as observed in human monocytes (Fig. 1g–i).

**Extended Data Fig. 7:**
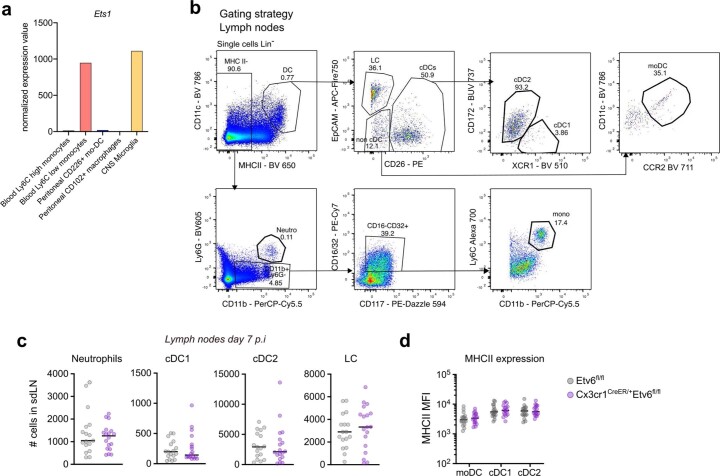
Analysis of immune cells in lymph nodes during EAE. **(a)** Expression of *Ets1* in blood monocytes, peritoneal mo-DC, peritoneal macrophages and Central Nervous System (CNS) microglia. RNA-seq data from ImmGen database. **(b-e)** EAE was induced by injection of MOG peptide. **(b)** EAE was induced in Cd11c-Etv6^Δ^ (KO) or WT littermates. Mean clinical score is shown. Bars represent SEM (n = 8-11 in 3 independent experiments). Peak clinical score. Median is shown. Each dot represents one mouse (n = 8-11 in 3 independent experiments). **(c-e)** Lymph nodes draining the site of MOG injection were analyzed 7 days after immunization. **(c)** Gating strategy for lymph node cell suspensions. Doublets and dead cells were excluded. Cells were separated into Langerhans cells (LC), cDC1, cDC2, monocytes (mono) and mo-DC. **(d)** Numbers of neutrophils, cDC1, cDC2 and LC in lymph nodes. Median is shown (n = 17-18 in 3 independent experiments). **(e)** MHC II expression on mo-DC, cDC1 and cDC2. Mean Fluorescence Intensity (MFI). Median is shown (n = 17-18 in 3 independent experiments).

Finally, we sought to apply our findings to a physiopathological setting. Mo-DCs have a deleterious role in EAE^8^, an animal model for multiple sclerosis (MS). In addition, IFN-β treatment improves disease symptoms and was reported to act primarily on myeloid cells^31^. Therefore, we hypothesized that Etv6 deletion in monocytes would ameliorate EAE outcome. We induced EAE in WT and Cx3cr1-Etv6^Δ^ mice by injection of myelin oligodendrocyte glycoprotein (MOG; Fig. 6g). Cx3cr1-Etv6^Δ^ mice showed less severe symptoms during the course of EAE (Fig. 6h) and reduced incidence (Fig. 6i). Of note, Cx3cr1-Etv6^Δ^ mice also target microglia. *Ets1*, which encodes an Etv6 antagonist^32^, is highly expressed in microglia (Extended Data Fig. 7a), suggesting that Etv6 action in microglia is naturally inhibited in WT mice. To directly address the potential role of microglial Etv6 during EAE, we induced *Etv6* deletion in Cx3cr1-Etv6^Δ^ mice by tamoxifen, then waited for 4 weeks to induce EAE (Fig. 6j). In this setting, only long-lived macrophages, including microglia, will remain deficient for *Etv6*, but short-lived monocytes will regain normal *Etv6* expression^33^. We found no difference in the development of EAE symptoms between WT and Cx3cr1-Etv6^Δ^ mice (Fig. 6k) and even increased the peak score in deficient mice (Fig. 6i). In addition, to exclude a role for Etv6 in cDCs in the ameliorated symptoms, we induced EAE in Cd11c-Etv6^Δ^ mice. We found no significant difference in EAE severity between Cd11c-Etv6^Δ^ mice and WT littermates (Extended Data Fig. 7b). These results confirm that *Etv6* deletion in monocytes, but not in microglia or cDCs, confers protection against severe EAE symptoms.

To understand the cellular mechanisms involved in ameliorated EAE outcome, we first analyzed DC populations in the lymph nodes draining the site of MOG injection during induction phase (7 d postimmunization) (Extended Data Fig. 7c). We found that mo-DCs were significantly decreased in the lymph nodes of Cx3cr1-Etv6^Δ^ mice (Fig. 6m,n), but not monocytes (Fig. 6n), neutrophils or other DC subsets (Extended Data Fig. 7d). It was recently shown that mo-DCs, but not cDC2 cells, are involved in the presentation of MOG antigen to CD4^+^ T cells in the lymph nodes^34^. MHC-II molecule expression by mo-DCs was unaffected by *Etv6* deletion (Extended Data Fig. 7e). We hypothesized that decreased mo-DC numbers in Cx3cr1-Etv6^Δ^ mice would reduce the induction of pathogenic CD4^+^ T cells. To test this, we assessed the presence in lymph nodes of MOG-specific CD4^+^ T cells using tetramer staining (Fig. 6o). We found that MOG-specific CD4^+^ T cells were significantly decreased in Cx3cr1-Etv6^Δ^ mice compared with WT mice, which can explain the reduced EAE symptoms in the central nervous system.

Collectively, these results confirm that Etv6 controls monocyte differentiation in vivo in mice during inflammation. We also identified Etv6 in monocytes as a therapeutic target for chronic inflammatory disorders such as MS.

## Discussion

In this work, we identified ETV3 and ETV6 as molecular regulators of the early stages of monocyte differentiation. We found that ETV3 and ETV6 act as repressors of mo-Mac fate commitment. We validated these observations in vivo, showing that mice deficient for Etv6 in monocytes display impaired mo-DC differentiation during inflammation. In addition, we found that Etv6 deletion in monocytes reduces the severity of EAE symptoms. Our findings allow a better understanding of the molecular control of monocyte fate decision and identify ETV6 as a therapeutic target in inflammatory disorders.

ETV3 and ETV6 are members of the Ets family of transcription factors. ETV6 is essential for hematopoietic stem cell survival^35^ and ETV3 was shown to regulate cell-cycle arrest^36^. However, their role in immune cells remains poorly understood. We have previously shown that ETV6 is expressed in DCs and facilitates the functional differentiation of cDC1 cells^32^. ETV3 was proposed to be a potential anti-inflammatory mediator downstream of IL-10 (ref. ^37^). In the present study, we identify ETV3 and ETV6 as key transcriptional regulators of mo-DC differentiation. Additional transcriptional repressors are probably involved in this process, because ETV3 or ETV6 transcriptional activity requires their association with corepressors. In particular, ETV6 has been shown to associate with IRF8 in a murine macrophage-like cell line^26^ and in mouse CD4 T cells^38^. Although IRF8 is essential for monocyte development from their progenitors^39,40^, whether it participates in mo-DC or mo-Mac differentiation is unknown. ETV6 has also been reported to associate in human PBMCs with NCOR2 (ref. ^24^), which regulates some of the IL-4-induced genes during human mo-DC differentiation^20^. In a human monocyte-like cell line, ETV3 was shown to associate with the repressor DP103, which interacts with the histone deacetylases HDAC2 and HDAC5 (ref. ^36^). Moreover, ETV6 recruits HDAC3 to the repressor complex in murine cell lines and human PBMCs^24,26,41^. Although a specific role for histone deacetylation in mo-DC fate commitment has not been described, it would be consistent with the fact that remodeling of histone acetylation occurs during monocyte differentiation^42^. Further work is needed to unravel the exact mechanism and molecular partners for the repression of ETV3 and ETV6 target genes in monocytes.

We have shown that ETV3 and ETV6 repress ISGs during monocyte differentiation and that ETV6 deletion in monocytes induces spontaneous ISG expression in vivo in mice. This is consistent with previous reports showing that ETV6 is involved in ISG repression in human PBMCs^24^ and binds to an IFN-stimulated response element in a reporter assay^26^. We also found that genes targeted by ETV3 versus ETV6 were only partially overlapping. This is in line with the observation that ETV7, another member of the Ets transcription repressor family, represses a subset of ISGs, but not all ISGs, in virus-exposed cells^43^. These observations suggest the existence of a specific pattern of target ISGs for each member of the ETV family.

We find that activation of the type I IFN pathway promotes mo-Mac differentiation in our culture system, where human monocytes are exposed to M-CSF, IL-4 and TNF. This is consistent with the finding that monocytes differentiated with granulocyte–macrophage (GM)-CSF and IL-4 in the presence of IFN-β display altered phenotype and functional features, suggesting impaired mo-DC differentiation, although the reorientation of their fate was not investigated^44^. In addition, IFN-γ and IL-4 have been shown to mutually inhibit each other’s programs in macrophages, via the crossrepression of STAT1 and STAT6 (ref. ^45^). In line with this idea, our results indicate that, when monocytes are exposed simultaneously to type I IFN and IL-4, the STAT1-induced program dominates that of STAT6. This suggests that STAT1 signaling would need to be repressed in monocytes to enable expression of the IL-4-dependent mo-DC differentiation program.

Monocyte-derived cells have been shown to play a central role in neuroinflammation. Mice deficient for CCR2 or its ligand, in which monocytes cannot exit the BM, are resistant to EAE or develop milder disease depending on strains^46–49^. In addition, blocking monocyte recruitment using a pharmacological inhibitor diminishes the incidence and severity of EAE^12^. Monocyte depletion after EAE onset also reduces inflammation and disease symptoms^13,46,50^. Mo-DCs and mo-Macs appear to play different roles during EAE. Mo-DCs are responsible for the presentation of myelin antigens during the induction phase of EAE^34^, and in the central nervous system stimulate pathogenic T_H_17 cells by secreting IL-23 (ref. ^8^). By contrast, mo-Macs display specific anti-inflammatory features during the resolution phase of EAE^51–53^. In patients with MS, monocyte recruitment is particularly increased in demyelinated areas^54^. Histological analysis also evidenced the presence around active MS lesions of myeloid cells that have a phenotype consistent with mo-DCs and are found to interact with numerous lymphocytes in situ^55^. Specific blocking of monocyte differentiation into mo-DCs, while preserving mo-Mac development, could therefore provide clinical benefits in neuroinflammation. Our results identify ETV6 as a candidate target to reorient monocyte fate decision for therapeutic strategies.

Collectively, our findings suggest that active repression of mo-Mac differentiation is required to allow monocyte differentiation toward mo-DCs. We propose a model whereby the mo-DC fate commitment in response to external cues (such as IL-4 and TNF) would require both the activation of the mo-DC differentiation program by factors, including IRF4, and the transcriptional repression of mo-Mac development by factors, including ETV3 and ETV6. Given the central role of mo-DCs in fueling pathogenic inflammation in numerous chronic inflammatory diseases, our work should have important implications for the therapeutic manipulation of monocyte differentiation.

## Methods

### Human samples

Buffy coats from healthy donors (both male and female donors) were obtained from Etablissement Français du Sang (Paris) in accordance with INSERM ethical guidelines. According to French Public Health Law (art L 1121-1-1, art L 1121-1-2), written consent and institutional review board approval are not required for human noninterventional studies.

### Mouse strains

Cx3Cr1-CreER were obtained from Jackson Laboratories (stock no. 021160). Cx3Cr1-CreER expresses the enhanced YFP from endogenous *Cx3cr1* promoter/enhancer elements. Etv6^flox/flox^ mice were obtained from H. Hock^35^. Cx3cr1-Etv6^Δ^ mice were generated by crossing Cx3Cr1-CreER^+/−^ mice with Etv6^flox/flox^ mice. Cd11c-Etv6^Δ^ mice have been described previously^32^. Cx3Cr1-CreER^−/−^ Etv6^flox/flox^ or CD11c-CreER^−/−^ Etv6^flox/flox^ littermates were used as WT controls, respectively. All mice were on a C57BL/6 background. Mice were maintained under specific pathogen-free conditions at the animal facility of Institut Curie or New York University School of Medicine, in accordance with institutional guidelines. Mice were housed in a 12 h light:12 h dark environment, with free access to water and food. Both male and female mice were used at age 7–9 weeks. All animal procedures were in accordance with the guidelines and regulations of the French Veterinary Department (authorization APAFIS no. 25217-2020042522586261 v.1) or the institutional animal care and use committee of New York University School of Medicine, and approved by the local ethics committee.

### Monocyte isolation and culture

PBMCs were prepared by centrifugation on a Ficoll gradient (Lymphoprep, STEMCELL). Blood CD14^+^ monocytes were isolated from healthy donors’ PBMCs by positive selection using magnetic beads (Miltenyi). Monocytes were 95–98% CD14^+^CD16^−^ as assessed by flow cytometry. Monocytes (2 × 10^6^ cells ml^−1^) were cultured for 5 d in RPMI–Glutamax medium (Gibco) supplemented with antibiotics (penicillin and streptomicin) and 10% fetal calf serum in the presence or absence of 100 ng ml^−1^ of M-CSF (Miltenyi), 5 ng ml^−1^ of IL-4 (Miltenyi) and 5 ng ml^−1^ of TNF-α (R&D Biotechne). Cytokines were added only at the start of the culture and the medium was not refreshed during the course of the culture. CD16^+^ or CD1a^+^ cell populations were isolated by cell sorting on a FACSAria instrument (BD Biosciences). In some experiments, monocytes were cultured in the presence of 100 ng ml^−1^ of M-CSF (Miltenyi), 5 ng ml^−1^ of IL-4 (Miltenyi) and 20 ng ml^−1^ of TNF (R&D Biotechne), or in the presence of IFN-α (recombinant human (rh) interferon-alpha 1b, Immunotools, catalog no. 11343596) or IFN-β (generated in-house by the platform of recombinant proteins of Institut Curie).

### Flow cytometry of human cells

Human cells were stained in phosphate-buffered saline (PBS) containing 0.5% human AB serum and 2 mM EDTA with APC anti-CD1a (BioLegend, clone HI149, dilution 1:300), FITC anti-CD16 (BioLegend, clone 3G8, dilution 1:200), PE-Cy7 anti-CD163 (BioLegend, clone GHI/61, dilution 1:100), PE anti-CD1b (eBioscience, clone eBioSN13, dilution 1:100). DAPI (Thermo Fisher Scientific, 100 ng ml^−1^) was added immediately before acquisition on a FacsVerse instrument (BD Biosciences) or MACSQuant (Miltenyi) instrument. Data were analyzed using FlowJo (v.10).

### Imaging flow cytometry

Cells were first stained with Live/Dead Aqua (Thermo Fisher Scientific) in PBS for 10 min at 4 °C. Then, cells were stained in PBS containing 0.5% human AB serum and 2 mM EDTA with anti-CD1a APC and anti-CD16 FITC for 30 min on ice. After washing, cells were fixed with paraformaldehyde 4% in PBS for 20 min at room temperature and permeabilized with Permeabilization Buffer (Fixation/Permeablization Kit, BD Biosciences) containing Fc block (Human TruStain FcX, BioLegend) and mouse serum (BioLegend) for 30 min on ice. Cells were then incubated with the primary antibody in permeabilization buffer, rabbit anti-ETV6/Tel (Novus Biologicals, NBP1-80695, dilution 1:1,000) or rabbit anti-ETV3 (Atlas Antibodies, HPA004794, dilution 1:1,000), at 4 µg ml^−1^ for 1 h at room temperature. Finally, cells were incubated with the secondary antibody anti-rabbit immunglobulin G (H + L) Alexa Fluor-594 (Molecular Probes, catalog no. A-11037, dilution 1:500) for 1 h at room temperature and then resuspended in staining buffer containing DAPI (Thermo Fisher Scientific, 50 ng ml^−1^). Samples were acquired in an Amnis ImageStream instrument (Luminex). Data were analyzed using the IDEAS software to obtain the similarity score between DAPI and Alexa Fluor-594 channels. Finally, data were exported to FlowJo for quantification and visualization.

### Phenotypic analysis of mouse tissues

For phenotypic analysis, Cx3cr1-Etv6^Δ^ mice and WT (Etv6^flox/flox^) littermates were treated with 5 mg of tamoxifen (Sigma-Aldrich) resuspended in corn oil (Sigma-Aldrich) by oral gavage for 3 consecutive days and sacrificed 5 d after the last treatment.

BMs were flushed from one leg and filtered using 40-μm cell strainers; 50 μl of of blood was used and incubated twice with red blood cell (RBC) lysis buffer (Sigma-Aldrich) for 5 min at room temperature. Spleens were cut into small pieces and incubated for 30 min at 37 °C in a digestion mix (RPMI containing 0.4 mg ml^−1^ of DNAse I (Sigma-Aldrich) and 0.5 mg ml^−1^ of collagenase D (Roche)). Spleen suspensions were then incubated with RBC lysis buffer for 5 min and filtered using 40-μm cell strainers. Peritoneal lavage was recovered by intraperitoneal injection of 5 ml of PBS.

### Flow cytometry of mouse tissues

Cells were stained in PBS containing bovine serum albumin (BSA) 0.5% and 2 mM EDTA for 30–45 min on ice. Antibodies used were anti-CD115 BUV395 (BD Bioscience, clone AFS98, dilution 1:100), anti-T cell receptor (TCR)-β BUV737 (BD Bioscience, clone H57-597, dilution 1:100), anti-CD172a BUV737 (BD Biosciences, clone P84, dilution 1:100), anti-Sca-1 BV421 (BioLegend, clone D7, dilution 1:100), anti-CD19 BV480 (BD Bioscience, clone 1D3, dilution 1/100), anti-TCR-β BV480 (BD Bioscience, clone H57-597, dilution 1:100), anti-NK1.1 BV480 (BD Bioscience, clone PK136, dilution 1:100), anti-Siglec-F BV480 (BD Bioscience, clone E50-2440, dilution 1:100), anti-XCR1 BV510 (BioLegend, clone ZET, dilution 1:100), anti-Ly6G BV510 (BioLegend, clone 1A8, dilution 1:300), anti-Ly6G BV605 (BioLegend, clone 1A8, dilution 1:300), anti-MHC-II BV650 (BioLegend, clone M5/114.15.2, dilution 1:100), anti-CCR2 BV711 (BD Bioscience, clone 475301, dilution 1:100), anti-CD11c BV785 (BioLegend, clone N418, dilution 1:100), anti-Ly6C BV785 (BioLegend, clone HK1.4, dilution 1:200), anti-CD45.1 BV785 (BioLegend, clone A20, dilution 1:200), anti-CD45.2 PE (BD Bioscience, clone 104, dilution 1:200), anti-CD26 PE (BioLegend, clone H194-112, dilution 1:100), anti-CD226 PE (BioLegend, clone 10E5, dilution 1:100), anti-CD11b PE da594 (BD Bioscience, clone M1/70, dilution 1:300), anti-CD117 PE da594 (BioLegend, clone 2B8, dilution 1:100), anti-CD11b PerCPCy5.5 (BD Biosciences, clone M1/70, dilution 1:300), anti-CD16/32 PE-Cy7 (BioLegend, clone 93, dilution 1:100), anti-F4/80 PE-Cy7 (BioLegend, clone BM8, dilution 1:50), anti-ESAM APC (BioLegend, clone 1G8/ESAM, dilution 1:100), anti-CD115 APC (BD Bioscience, clone AFS98, dilution 1:100), anti-TIM4 APC (BioLegend, clone RMT4-54, dilution 1:100), anti-Ly6C Alexa 700 (BioLegend, clone HK1.4, dilution 1:200), anti-EpCAM APCFire750 (BioLegend, clone G8.8, dilution 1:400), anti-MHC-II APC Cy7 (BioLegend, clone M5/114.15.2, dilution 1:200) and anti-intercellular adhesion molecule (ICAM)-2 Biotin (BioLegend, clone 3C4, dilution 1:100), followed by streptavidin BV421 (Invitrogen, dilution 1:100). Antibody panels for the different tissues can be found in Supplementary Table 4. After washing, cells were resuspended in staining buffer containing DAPI (100 ng ml^−1^). Cells were acquired on a ZE5 flow cytometer (BioRad). Supervised analysis was performed using FlowJo software.

### The cMoP differentiation assay

Cx3cr1-Etv6^Δ^ mice and WT littermates were treated with 5 mg of tamoxifen resuspended in corn oil by oral gavage for 3 consecutive days (days 0–2). On day 5, cMoPs were sorted from the BM on a FACSAria Fusion instrument (BD Biosciences). The cMoPs were gated as CD19l^−^TCRβ^−^Ly6G^−^CD11b^−^CD115^+^CD16/32^high^CD117^+^Ly6C^+^. Sorted cells (20,000–30,000 cells per well in 96-well plates) were cultured for 3 d in RPMI–Glutamax medium supplemented with sodium pyruvate (1 mM), Hepes (10 mM), antibiotics (penicillin and streptomycin) and 10% FCS in the presence of 50 ng ml^−^ of hrM-CSF. Cells were analyzed by flow cytometry.

### ShRNA interference

ShRNA (all from Sigma-Aldrich) against ETV3 (sh1: NM_005240-TRCN0000013930; sh2: NM_005240-TRCN0000013931; sh3: NM_005240-TRCN0000013932), ETV6 (sh1: NM_001987- TRCN0000003853; sh2: NM_001987-TRCN0000003854; sh3: NM_001987-TRCN0000003855), or nontargeting control shRNA (MISSION shRNA SHC002) were in the LKO.1-puro vector (MISSION, Sigma-Aldrich). Viral particles were produced by transfection of 293FT cells (American Type Culture Collection) in 6-well plates with 3 mg of DNA and 8 µl of TransIT-293 (Mirus Bio) per well: for vesicular stomatitis virus G (VSV-G) pseudotyped SIVmac VLPs, 0.4 mg of cytomegalovirus (CMV)–VSV-G and 2.6 mg of pSIV3^+^; for shRNA vectors, 0.4 mg of CMV–VSV-G, 1 mg of psPAX2 and 1.6 mg of LKO1puro-derived shRNA vector. Then, 1 d after 293FT cell transfection, medium was replaced by fresh culture medium. Viral supernatants were harvested 1 d later and filtered through 0.45-μm filters. Freshly isolated CD14^+^ monocytes were infected with viral particles containing the control vector or individual shRNA vectors and cultured as above. Puromycin (InvivoGen) was added 2 d later (2 mg ml^−1^). At day 5, cells were harvested for analysis.

### Overexpression

ETV6 complementary DNA was cloned in a pTRIP-SFFV-BFP-P2A vector. Viral particles were produced by transfection of 293FT cells in 6-well plates with 3 mg of DNA and 8 µl of TransIT-293 (Mirus Bio) per well: for VSV-G pseudotyped SIVmac VLPs, 0.4 mg of CMV–VSV-G and 2.6 mg of pSIV3^+^; for ETV6 vectors, 0.4 mg of CMV–VSV-G, 1 mg of psPAX2 and 1.6 mg of pTRIP-SFFV-BFP-P2A-derived vector. Then 1 d after 293FT cell transfection, medium was replaced by fresh culture medium. Viral supernatants were harvested 1 d later and filtered through 0.45-μm filters. Freshly isolated CD14^+^ monocytes were infected with viral particles containing the control pTRIP-SFFV-BFP-P2A or pTRIP-SFFV-BFP-P2A-ETV6 vector. At day 5, cells were harvested for immunoblotting or FACS analysis.

### Quantitative PCR

For the analysis of BM monocytes, monocytes were purified with EasySep mouse monocyte isolation kit (STEMCELL) according to the manufacturer’s recommendations. Cells from the peritoneal lavage or the spleen were sorted on a FACSAria Fusion instrument before lysis.

Cells were harvested and lysed in RLT buffer (QIAGEN). RNA extraction was carried out using the RNAeasy micro kit (QIAGEN) according to the manufacturer’s instructions. Total RNA was retro-transcribed using the superscript II polymerase (Invitrogen), in combination with random hexamers, oligo-dT and dNTPs (Promega). Transcripts were quantified by RT–PCR on a 480 LightCycler instrument (Roche). Reactions were carried out in 10 μl, using a master mix (Eurogentec), with the following Taqman Assays primers (Merk), for human samples: B2M (catalog no. Hs99999907_m1), RPL34 (catalog no. Hs00241560_m1), HPRT1 (catalog no. Hs02800695_m1), ETV3 (catalog no. Hs01051028_g1), ETV6 (catalog no. Hs00231101_m1), MX1 (catalog no. Hs00895608_m1), IFIT3 (catalog no. Hs00155468_m1), CXCL10 (catalog no. Hs00895608_m1), MAFB (catalog no. Hs00271378_s1), PPARG (catalog no. Hs01115513_m1) and ZNF366 (catalog no. Hs00403536_m1); and for mouse samples**:** Gapdh (catalog no. Mm99999915_g1), B2m (catalog no. Mm00437762_m1), Polr2a (catalog no. Mm00839502_m1), Etv6 (catalog no. Mm01261325_m1), Isg15 (catalog no. Mm01705338_s1), Mx1 (catalog no. Mm00487796_m1), Cxcl10 (catalog no. Mm00445235_m1), Ly6a (catalog no. Mm00726565_s1), Ifna1-Ifna5-Ifna6 (catalog no. Mm03030145_gH), Ifnb1 (catalog no. Mm00439552_s1), Ifng (catalog no. Mm01168134_m1) and Ifnl2-Ifnl3 (catalog no. Mm04204158_gH). The second derivative method was used to determine each Cp and the expression of genes of interest relative to the housekeeping genes was quantified: B2M (catalog no. HS00187842_m1), HPRT (catalog no. Hs02800695_m1) and RPL34 (catalog no. Hs00241560_m1) for humans; and Gapdh (catalog no. Mm99999915_g1), B2M (catalog no. Mm00437762_m1) and Polr2a (Mm00839502_m1) for mice.

### Immunoblotting

Cells were lysed in radioimmunoprecipitation assay (RIPA) buffer (Fisher Thermo Scientific) supplemented with complete Mini EDTA-free protease inhibitor cocktail (Roche), at 1 × 10^6^ cells in 100 μl of lysis buffer. Postnuclear lysates were resolved by sodium dodecylsulfate–polyacrylamide gel electrophoresis using 4–15% BisTris NuPAGE gels (Invitrogen) and proteins were transferred to membranes (Immunoblot PVDF membranes, BioRad). Membranes were stained with primary antibodies against ETV6/Tel (Novus Biologicals, catalog no. NBP1-80695, 0.4 μg ml^−1^), ETV3 (Atlas Antibodies, catalog no. HPA004794, 0.4 μg ml^−1^), GP96 (Novus Biologicals, clone 9G10, 0.4 μg ml^−1^) or actin (Millipore, clone C4, 0.4 μg ml^−1^), followed by horeseradish peroxidase-conjugated secondary antibodies (Jackson Immunoresearch, dilution 1:10,000). Some membranes were incubated with Re-blot Plus buffer (Millipore). Densitometry quantification was performed using Fiji (v.2.9).

### ScRNA-seq

Monocytes were purified from two individual donors. Cells were barcoded per donor (donors A and B) using TotalSeq-anti-human Hashtag antibody (catalog nos. A0251 and A02052, respectively; BioLegend) according to the manufacturer’s instruction. Barcoded cells were counted and mixed in a 1:1 ratio. Single-cell suspension was loaded into 10x Genomics Chromium. Libraries were prepared as per the manufacturer’s protocol (Chromium Single Cell 3′ Reagent Kits v.3 protocol) and sequenced on an Illumina NovaSeq sequencer according to the 10x Genomics recommendations (paired-end reads) to a depth of approximately 50,000 reads per cell.

Initial processing was performed using Cell Ranger (v.3.1.0) and subsequent analysis with Seurat v.4.0 workflow^56^. Hashtag demultiplexing was performed using the function HTODemux() and positive.quantile = 0.99. Cells with >20% of mitochondrial genes or genes expressed in <3 cells were filtered out. Graph-based clustering, visualization and DEG analyses were performed using Seurat v.4.0. For clustering analysis, FindNeighbors() and FindClusters() functions of the Seurat package were used with the first 50 significant principal components (PCs) and a resolution of 1.3, respectively. For identification of DEGs, FindMarkers or FindAllMarkers function (test.use = ‘t’, logfc.threshold = log[0.25]) were used based on normalized data. DEGs with *P*_adj_ > 0.05 were filtered out. Data have been deposited in the National Center for Biotechnology Information’s (NCBI’s) Gene Expression Omnibus (GEO) and can be accessed at accession no. GSE211603.

### RNA-seq library preparation

Monocytes were cultured for 3 d in the presence of 100 ng ml^−1^ of M-CSF, 5 ng ml^−1^ of IL-4 and 20 ng ml^−1^ of TNF. Total RNA was extracted using the RNAeasy minikit (QIAGEN) including on-column DNase digestion according to the manufacturer’s protocol. The integrity of the RNA was confirmed in BioAnalyzer using RNA 6000 Pico kit (Agilent Technologies) (8.8 < RNA integrity no. (RIN) < 10). Libraries were prepared according to Illumina’s instructions accompanying the TruSeq Stranded mRNA Library Prep Kit (Illumina). RNA, 500 ng, was used for each sample. Library length profiles were controlled using the LabChip GXTouchHT system (Perkin Elmer). Sequencing was performed in four sequencing units of NovaSeq 6000 (Illumina) (100–593 nt length reads, paired end) with an average depth of 40 × 10^6^ clusters per sample. RNA-seq data discussed in this publication have been deposited in the NCBI’s GEO and can be accessed at accession no. GSE188982.

### RNA-seq data analysis

Genome assembly was based on the Genome Reference Consortium (hg38). The quality of the RNA-seq data was assessed using FastQC (v.0.11.8)^57^. Reads were aligned to the transcriptome using STAR (v.2.6.1)^58^. DEG analysis was performed using DESeq2 (v.1.22.2) with the design ‘donor + group’^59^. Genes with a low number of counts (<10) were filtered out. DEGs were identified based on *P*_adj_ < 0.05 and absolute log_2_(FC) > 0.5. Volcano plots were generated with EnhancedVolcano^60^. Transcription factor activity was calculated using Dorothea regulons (v.1.0.1) and VIPER (v.1.3)^22^.

### GSEA

GSEA^61^ was performed using the GSEA software (v.4.0.3) with the default parameters, except for the number of permutations that we fixed at *n* = 1,000. The count matrix from RNA-seq studies was first normalized using DESeq2. Gene signatures of blood monocytes, mo-DCs and mo-Macs were designed from microarray data^18^.

### Analysis of public RNA-seq data

ScRNA-seq data from healthy controls and patients with the ETV6^P214L^ mutation^24^ were downloaded from BioProject, accession no. PRJNA657295 and processed using Cell Ranger (v.2.1.0) using the human reference genome (hg38). Count matrices were then integrated and analyzed using Seurat v.4. Cells with <200 genes detected or percentage of mitochondrial genes >15%, as well as genes detected in <3 cells, were excluded from the analysis. We used a reference-mapping approach to annotate cell labels in the query dataset using the MapQuery function of Seurat v.4. We first projected each query cell on to a previously computed Uniform Manifold Approximation and Projection (UMAP) visualization of the reference dataset^56^ (Extended Data Fig. 2a,b) and then, we subset the CD14^+^ and CD16^+^ monocytes. The dimensionality reduction, PC analysis and UMAP projection were performed in this subset (Extended Data Fig. 2c). Differential signature enrichment was calculated using Student’s *t*-test comparing the signature score in WT versus P214L patients.

RNA-seq-normalized expression values for *Ets1* in mouse blood monocytes (Ly6C^+^ and Ly6C^−^ monocytes), peritoneal mo-DCs (CD226^+^MHCII^+^F4/80^low^), peritoneal macrophages (CD226^−^MHCII^−^CD102^+^F4/80^+^) and central nervous system microglia were downloaded from the ImmGen database (www.immgen.org).

### ChIP coupled to sequencing

Monocytes from 2 individual donors were cultured for 3 d in the presence of 100 ng ml^−1^ of M-CSF, 5 ng ml^−1^ of IL-4 and 20 ng ml^−1^ of TNF. ChIP was performed from 80 × 10^6^ monocytes as the starting material, using the ChIP-IT High Sensitivity kit (Active Motif) following the manufacturer’s instructions. Monocytes were homogenized using a 7-ml Dounce glass homogenizer (Clinisciences). Sonication was performed using a Bioruptor Pico instrument (Diagenode) using 20 cycles (30 s on and 30 s off). Chromatin was immunoprecipitated using antibodies against ETV3 (ref. A303-737A, polyclonal, Bethyl) or ETV6 (clone R1092.1.1A9, CDI Laboratories Inc.). Libraries were prepared according to Illumina’s instructions accompanying the TruSeq ChIPSeq Library Prep Kit (Illumina). DNA, 4–6 ng, was used for each sample. Sequencing was performed in one sequencing unit of NovaSeq 6000 (Illumina) (100- to 593-nt length reads, paired end).

The quality of the sequencing data was assessed using FastQC^57^. Reads were aligned to the genome using BWA-Mem^62^. Peak calling was performed with MACS2 (ref. ^63^) using default parameters for each donor and the corresponding input. Final peaks were obtained by intersecting both donors using BEDTools^64^ with the parameter ‘-f 0.5’. Peak assignment was performed on the intersected bed file using the annotatePeaks function of HOMER^65^, with the genome ‘hg38’ as a reference. Known motif enrichment analysis was performed using HOMER with the findMotifsGenome function, and the parameters ‘hg38 -size given --mask’. ChIP–seq data have been deposited in NCBI’s GEO and can be accessed at accession no. GSE211604.

### Experimental peritonitis

Cx3cr1-Etv6^Δ^ mice and WT (Etv6^flox/flox^) littermates were treated with 5 mg of tamoxifen resuspended in corn oil by oral gavage for 3 consecutive days (days 0–2). On day 5, mice received a fourth gavage of tamoxifen and were injected intraperitoneally with 1 ml of 3.8% brewer’s thioglycolate medium (Sigma-Aldrich). Mice were analyzed 3 d after thioglycolate injection.

### Monocyte adoptive transfer

Cx3cr1-Etv6^Δ^ mice and WT littermates were treated with 5 mg of tamoxifen resuspended in corn oil by oral gavage for 3 consecutive days (days 0–2). On day 5, monocytes were isolated from BM using the EasySep Mouse Monocyte Isolation Kit according to the manufacturer’s instructions. Between 0.7 × 10^6^ and 1 × 10^6^ monocytes were injected intraperitoneally in CD45.1 C57BL/6 mice that had been injected 18 h before with 1 ml of 3.8% brewer’s thioglycolate medium. Peritoneal lavage was analyzed by flow cytometry 3 d after monocyte injection.

### Experimental autoimmune encephalomyelitis

Cx3cr1-Etv6^Δ^ mice and WT (Etv6^flox/flox^) littermates were treated with 5 mg of tamoxifen resuspended in corn oil by oral gavage twice a week, starting 1 week before immunization. In some experiments (deletion in microglia), Cx3cr1-Etv6^Δ^ mice and WT littermates were treated with tamoxifen twice, then rested for 4 weeks before immunization. In some experiments, Cd11c-Etv6^Δ^ mice and WT (Etv6^flox/flox^) littermates were used. Mice were immunized subcutaneously in the back with 100 µg of the MOG35–55 peptide (sb-PEPTIDE) emulsified in incomplete Freud’s adjuvant (Invivogen) supplemented with 4 mg ml^−1^ of desiccated *Mycobacterium* *tuberculosis* (Sigma-Aldrich, catalog no. H37RA). Mice were injected intraperitoneally with 200 ng of pertussis toxin from *Bordetella pertussis* (Calbiochem) at days 0 and 2 after immunization. Mice were examined daily for clinical signs. In agreement with the local ethics committee, mice were scored as follows: 0, healthy; 0.5, tail weakness; 1, limp tail; 1.5, tail paralysis and hindlimb weakness; 2, tail paralysis and limping of one hindlimb; 2.5, tail paralysis and limping of both hindlimbs; 3, paralysis of tail and both hindlimbs; and 3.5, paralysis of tail and both hindlimbs and weakness in forelimbs. A score of 3 was predefined as the humane endpoint of the experiment.

### Lymph node cell analysis during EAE

Inguinal lymph nodes were collected 7 d post-MOG immunization. For flow cytometry of DCs, lymph nodes were cut into small pieces and incubated for 30 min at 37 °C in digestion mix: RPMI containing 0.5 mg ml^−1^ of DNAse I and 0.5 mg ml^−1^ of collagenase D. Cell suspensions were then filtered using 40-μm cell strainers. An antibody panel can be found in Supplementary Table 1. For tetramer staining, lymph nodes were dissociated by forcing through a 40-μm cell strainer. Cells were incubated for 3 h at 37 °C in RPMI containing 10% FCS in the presence of phycoerythrin (PE)-conjugated MOG tetramer (I-A(b) GWYRSPFSRVVH, 2.7 mg ml^−1^) or control tetramer (I-A(b) PVSKMRMATPLLMQA, 2.7 mg ml^−1^) (both obtained from the National Institutes of Health (NIH) tetramer core facility). After washing, cells were stained with anti-CD8 BUV395 (BD Bioscience, clone H35-17.2), T cell receptor (TCR)-β BUV737 (BD Bioscience, clone H57-597), CD19 BV480 (BD Bioscience, clone 1D3), CD4 PerCPCy5.5 (BD Bioscience, clone RM4-5) and CD11b PeCy7 (BD Bioscience, clone M1/70).

### Statistical analysis

Sample-size calculations were performed using InVivoStat (v.4.2). For experiments on human cells, based on our previous results, we estimated the interdonor variability to 100% coefficient of variation (CV) in these experiments. In these conditions, *n* = 5 is sufficient for 70% power and *n* = 6 for 80% power to detect biologically significant results with a 5% significance level. Therefore, we used *n* = 5–6 as a minimum group size for these experiments. For animal experiments, based on our previous results, we estimated the interanimal variability to 50% CV in these experiments. In these conditions, *n* = 9 is sufficient for 80% power to detect a doubling of response between groups. Therefore, we used a minimum of nine mice in these experiments. For RNA-seq analysis, we used five samples per group, based on the literature for optimal group size in RNA-seq experiments^66^. Wilcoxon’s matched-paired test, Mann–Whitney *U*-test and unpaired Student’s *t*-test were performed using Prism v.9 (GraphPad Software). All statistical tests were two sided. Data distribution was assumed to be normal but this was not formally tested. Statistical details for each experiment can be found in the corresponding figure legend; *n* corresponds to the number of biological replicates (individual human donors or individual mice). No animal or data points were excluded. Data collection was performed blinded and randomized. Blinding was not possible for EAE because measurements were performed longitudinally for each mouse. Blinding was not performed for immunoblots to allow silenced samples to be presented side by side.

### Reporting summary

Further information on research design is available in the Nature Portfolio Reporting Summary linked to this article.

## Online content

Any methods, additional references, Nature Portfolio reporting summaries, source data, extended data, supplementary information, acknowledgements, peer review information; details of author contributions and competing interests; and statements of data and code availability are available at 10.1038/s41590-022-01374-0.

## Supplementary information

Reporting Summary

Supplementary Table 1 List of DEGs between control and ETV3 silencing in human monocytes.

Supplementary Table 2 List of DEGs between control and ETV6 silencing in human monocytes.

Supplementary Table 3 List of peaks identified in ChIP–seq analysis for ETV3 and ETV6.

Supplementary Table 4 Antibody panels used in the study.

## Data Availability

Sequencing data have been deposited in the GEO under accession nos. GSE188982 (RNA-seq), GSE211603 (scRNA-seq) and GSE211604 (ChIP–seq). Publicly available data were obtained from the Immgen database (www.immgen.org), BioProject (accession no. PRJNA657295) and the reference genome hg38 from Ensembl (www.ensembl.org). All other data are in the article and supplementary files are available from the corresponding author upon reasonable request. Source data are provided with this paper.

## Source data

Source Data Fig. 1 Uncropped immunoblots from Fig. 1. The parts of the gels that were used for the figure panels are highlighted with a red box.
Source Data Fig. 4 Uncropped immunmoblots from Fig. 4. The parts of the gels that were used for the figure panels are highlighted with a red box.
