# Supplementary material for: ETV3 and ETV6 enable monocyte differentiation into dendritic cells by repressing macrophage fate commitment
Source: Nat Immunol. 2022 Dec 21;24(1):84–95. doi: 10.1038/s41590-022-01374-0 (PMC9810530; doi:10.1038/s41590-022-01374-0)
Supplement: Supplementary file 1 — Reporting Summary [file 41590_2022_1374_MOESM1_ESM.pdf]

nature portfolio

Corresponding author(s): Elodie Segura

Last updated by author(s): Oct 5, 2022

Reporting Summary

Nature Portfolio wishes to improve the reproducibility of the work that we publish. This form provides structure for consistency and transparency in reporting. For further information on Nature Portfolio policies, see our Editorial Policies and the Editorial Policy Checklist.

Statistics

For all statistical analyses, confirm that the following items are present in the figure legend, table legend, main text, or Methods section.

n/a	Confirmed	
☐	☒	The exact sample size (*n*) for each experimental group/condition, given as a discrete number and unit of measurement
☐	☒	A statement on whether measurements were taken from distinct samples or whether the same sample was measured repeatedly
☐	☒	The statistical test(s) used AND whether they are one- or two-sided<br>*Only common tests should be described solely by name; describe more complex techniques in the Methods section.*
☐	☒	A description of all covariates tested
☐	☒	A description of any assumptions or corrections, such as tests of normality and adjustment for multiple comparisons
☐	☒	A full description of the statistical parameters including central tendency (e.g. means) or other basic estimates (e.g. regression coefficient) AND variation (e.g. standard deviation) or associated estimates of uncertainty (e.g. confidence intervals)
☐	☒	For null hypothesis testing, the test statistic (e.g. *F*, *t*, *r*) with confidence intervals, effect sizes, degrees of freedom and *P* value noted<br>*Give P values as exact values whenever suitable.*
☒	☐	For Bayesian analysis, information on the choice of priors and Markov chain Monte Carlo settings
☒	☐	For hierarchical and complex designs, identification of the appropriate level for tests and full reporting of outcomes
☒	☐	Estimates of effect sizes (e.g. Cohen's *d*, Pearson's *r*), indicating how they were calculated

Our web collection on statistics for biologists contains articles on many of the points above.

Software and code

Policy information about availability of computer code

Data collection	No software was used for data collection
Data analysis	Flow cytometry data was analyzed with FlowJo (v10). Imaging flow cytometry was analyzed with IDEAS software. Densitometry quantification was performed using Fiji (v2.9). scRNA-seq from monocytes was analyzed using CellRanger (v3.1.0) and processed using Seurat (v4.0). GSEA was performed using the GSEA software (version 4.0.3) with the default parameters, except for the number of permutations that we fixed at n=1000. Single-cell RNA-Seq data from public data (healthy controls and patients with the ETV6P214L mutation) was downloaded from BioProject PRJNA657295 and was processed with Cell Ranger (v2.1.0) using the human reference genome (hg38). Count matrices were then integrated and analyzed using Seurat v4. For RNA-seq analysis, genome assembly was based on the Genome Reference Consortium (hg38). Quality of RNA-seq data was assessed using FastQC (v0.11.8). Reads were aligned to the transcriptome using STAR (v2.6.1a_08-27). Differential gene expression analysis was performed using DESeq2 (v1.22.2). Transcription factor activity was calculated using Dorothea (v1.0.1) and VIPER (v1.3). Volcano plots were generated with EnhancedVolcano (v1.14). ChIP-seq data was analyzed using BWA-Mem (v0.77.11), MACS2 (v2.2.7.1), BEDTools (v2.30) and HOMER (v4.8). Sample-size calculation was performed using InVivoStat (v4.2). Statistical analysis was performed using Prism v9 (GraphPad Software).

For manuscripts utilizing custom algorithms or software that are central to the research but not yet described in published literature, software must be made available to editors and reviewers. We strongly encourage code deposition in a community repository (e.g. GitHub). See the Nature Portfolio guidelines for submitting code & software for further information.

Data

Policy information about availability of data

All manuscripts must include a data availability statement. This statement should provide the following information, where applicable:

- Accession codes, unique identifiers, or web links for publicly available datasets
- A description of any restrictions on data availability
- For clinical datasets or third party data, please ensure that the statement adheres to our policy

All datasets generated and/or analyzed during the study are referenced in this article. Sequencing data generated during the study can be accessed in GEO under the numbers GSE188982 (RNA-seq), GSE211603 (scRNA-seq) and GSE211604 (ChIP-seq). Source data or supplementary information are available from the corresponding author upon reasonable request. Publicly available data were obtained from Immgen database (www.immgen.org), from BioProject PRJNA657295 and the reference genome hg38 from Ensembl (www.ensembl.org).

Field-specific reporting

Please select the one below that is the best fit for your research. If you are not sure, read the appropriate sections before making your selection.

☒ Life sciences ☐ Behavioural & social sciences ☐ Ecological, evolutionary & environmental sciences

For a reference copy of the document with all sections, see nature.com/documents/nr-reporting-summary-flat.pdf

Life sciences study design

All studies must disclose on these points even when the disclosure is negative.

Sample size	Sample-size calculation was performed using InVivo stat software (v4.2). For experiments on human cells, based on our previous work in this system (DOI: 10.1073/pnas.2109225118, DOI: 10.1016/j.immuni.2017.08.016), we estimated the inter-donor variability to 100% CV in these experiments. In these conditions, n=5 is sufficient for having 70% power and n=6 for having 80% power to detect biologically significant results with a 5% significance level. Therefore, we used n=5-6 as a minimum group size for experiments using human monocytes. For animal experiments, based on our previous work (DOI: 10.1016/j.immuni.2017.08.016), we estimated the inter-animal variability to 50% CV in these experiments. In these conditions, n=9 is sufficient for having 80% power to detect a doubling of response between groups. Therefore we used a minimum of 9 mice in these experiments. For RNA-seq analysis, we used 5 samples per group, based on the literature for optimal group size in RNA-seq experiments (DOI: 10.1261/rna.053959.115). For scRNA-seq and ChIP-seq, we used 2 biological replicates, without re-determining group size.
Data exclusions	No data was excluded.
Replication	Experiments were replicated independently. The number of biological replicates and of independent experiments is indicated in each legend.
Randomization	For all experiments in this manuscript, samples or animals were allocated to experiments and experimental groups randomly.
Blinding	For all experiments, investigators were blinded during data collection by giving each sample analyzed an arbitrary number. Blinding was not possible for analyzing Experimental Autoimmune Encephalomyelitis, because measurements were performed longitudinally for each mouse. Blinding was not possible when preparing gels for Western Blots to allow relevant samples to be presented side-by-side on the gel.

Reporting for specific materials, systems and methods

We require information from authors about some types of materials, experimental systems and methods used in many studies. Here, indicate whether each material, system or method listed is relevant to your study. If you are not sure if a list item applies to your research, read the appropriate section before selecting a response.

Materials & experimental systems

n/a	Involved in the study	
☐	☒	Antibodies
☐	☒	Eukaryotic cell lines
☒	☐	Palaeontology and archaeology
☐	☒	Animals and other organisms
☐	☒	Human research participants
☒	☐	Clinical data
☒	☐	Dual use research of concern

Methods

n/a	Involved in the study	
☐	☒	ChIP-seq
☐	☒	Flow cytometry
☒	☐	MRI-based neuroimaging

Antibodies

Antibodies used	For human cells, antibodies for flow cytometry are : APC anti-CD1a (Biolegend, clone HI149, dilution 1/300), FITC anti-CD16 (Biolegend, clone 3G8, dilution 1/200), PE-Cy7 anti-CD163 (Biolegend, clone GHI/61, dilution 1/100), PE anti-CD1b (eBioscience, clone eBioSN13, dilution 1/100). For mouse cells, antibodies for flow cytometry were anti-CD115 BUV 395 (BD Bioscience, clone AFS98, dilution 1/100), anti-TCRβ BUV737 (BD Bioscience, clone H57-597, dilution 1/100), anti-CD172a BUV737 (BD Biosciences, clone P84, dilution 1/100), anti-Sca1 BV421 (Biolegend, clone D7, dilution 1/100), anti-CD19 BV480 (BD Bioscience clone 1D3, dilution 1/100), anti-TCRβ BV480 (BD Bioscience, clone H57-597, dilution 1/100), anti-NK1.1 BV480 (BD Bioscience, clone PK136, dilution 1/100), anti-SiglecF BV480 (BD Bioscience, clone E50-2440, dilution 1/100), anti-XCR1 BV510 (Biolegend, clone ZET, dilution 1/100), anti-Ly6G BV510 (Biolegend, clone 1A8, dilution 1/300), anti-Ly6G BV605 (Biolegend, clone 1A8, dilution 1/300), anti-MHC II BV650 (Biolegend, clone M5/114.15.2, dilution 1/100), anti-CCR2 BV711 (BD Bioscience, clone 475301, dilution 1/100), anti-CD11c BV785 (Biolegend, clone N418, dilution 1/100), anti-Ly6C BV785 (Biolegend, clone HK1.4, dilution 1/200), anti-CD45.1 BV785 (Biolegend, clone A20, dilution 1/200), anti-CD45.2 PE (BD Bioscience, clone 104, dilution 1/200), anti-CD26 PE (Biolegend, clone H194-112, dilution 1/100), anti-CD226 PE (Biolegend, clone 10E5, dilution 1/100), anti-CD11b PE da594 (BD Bioscience, clone M1/70, dilution 1/300), anti-CD117 PE da594 (Biolegend, clone 2B8, dilution 1/100), anti-CD11b PerCPCy5.5 (BD Biosciences, clone M1/70, dilution 1/300), anti-CD16/32 PECy7 (Biolegend, clone 93, dilution 1/100), anti-F4/80 PECy7 (Biolegend, clone BM8, dilution 1/50), anti-ESAM APC (Biolegend, clone 1G8/ESAM, dilution 1/100), anti-CD115 APC (BD Bioscience, clone AFS98, dilution 1/100), anti-TIM4 APC (Biolegend, clone RMT4-54, dilution 1/100), anti-Ly6C Alexa 700 (Biolegend, clone HK1.4, dilution 1/200), anti-EpCAM APCFire750 (BioLegend, clone G8.8, dilution 1/400), anti-MHC II APC Cy7 (Biolegend, clone M5/114.15.2, dilution 1/200) and anti-ICAM2 Biotin (Biolegend, clone 3C4, dilution 1/100) followed by Streptavidin BV421 (Invitrogen, dilution 1/100). Antibody panels for the different tissues can be found in Supplementary Table 1. For Western blots, membranes were stained with primary antibodies against ETV6/Tel (Novus Biologicals, NBP1-80695, 0.4 µg/mL), ETV3 (Atlas Antibodies, HPA004794, 0.4 µg/mL), GP96 (Novus Biologicals, clone 9G10, 0.4 µg/mL), or actin (Millipore, clone C4, 0.4 µg/mL), followed by HRP-conjugated secondary antibodies (Jackson Immunoresearch, dilution 1/10000). For imaging flow cytometry, antibodies used are : rabbit anti-ETV6/Tel (Novus Biologicals, NBP1-80695, dilution 1/1000) or rabbit anti- ETV3 (Atlas Antibodies, HPA004794, dilution 1/1000) and the secondary antibody anti-Rabbit IgG (H+L) Alexa 594 (Molecular Probes, #A-11037, 1/500). Chromatin was immunoprecipitated using antibodies against ETV3 (ref A303-737A, polyclonal, Bethyl) or ETV6 (clone R1092.1.1A9, CDI Laboratories Inc).
Validation	All antibodies were validated by the manufacturer, for the species and the specific application for which they were used in the manuscript (flow cytometry, Western Blot, ChIP). For imaging flow cytometry, we used antibodies validated for Western Blot.

Eukaryotic cell lines

Policy information about cell lines

Cell line source(s)	293FT cells were from ATCC
Authentication	293FT cells were authenticated by the supplier.
Mycoplasma contamination	The cell line was routinely tested for mycoplasma and was free of contamination.
Commonly misidentified lines (See ICLAC register)	No commonly misidentified cell lines were used in this study.

Animals and other organisms

Policy information about studies involving animals; ARRIVE guidelines recommended for reporting animal research

Laboratory animals	Cx3Cr1-CreER were obtained from Jackson Laboratories (Stock # 021160). Cx3Cr1-CreER express the enhanced yellow fluorescent protein (EYFP) from endogenous Cx3cr1 promoter/enhancer elements. Etv6flox/flox mice were obtained from H.Hock. Cx3cr1-Etv6Δ were generated by crossing Cx3Cr1-CreER+/- mice with Etv6flox/flox mice. CD11c-Etv6Δ have been previously described (DOI: 10.1084/jem.20172323). Cx3Cr1-CreER-/- Etv6flox/flox or CD11c-CreER-/- Etv6flox/flox littermates were used as WT controls, respectively. All mice were on C57BL/6 background. Mice were maintained under specific pathogen-free conditions at the animal facility of Institut Curie or New York University School of Medicine, in accordance with institutional guidelines. Both male and female mice were used at age 7-9 weeks.
Wild animals	No wild animals were used in this study.
Field-collected samples	No field-collected samples were used in this study.
Ethics oversight	All animal procedures were in accordance with the guidelines and regulations of the French Veterinary Department (authorization APAFIS #25217-2020042522586261 v1), or Institutional animal Care and Use Committee of New York University School of Medicine and approved by the local ethics committee.

Note that full information on the approval of the study protocol must also be provided in the manuscript.

Human research participants

Policy information about studies involving human research participants

Population characteristics	Buffy coats from healthy donors (both male and female donors, between 18 and 70 years old) were obtained from

Population characteristics	Etablissement Français du Sang (Paris) in accordance with INSERM ethical guidelines. All blood samples were anonymized before delivery.
Recruitment	No specific recruitment parameters were used, besides standard procedure for recruitment at the French blood bank (Etablissement français du sang): age 18-70 years old, both male and female, weight over 50kg, sero-negative for HIV, HBV and HVC, never suffered from cancer, absence of current treatment for chronic diseases, absence of ongoing infection, never received a blood transfusion or organ transplant, not having given birth in the past 6 months, not having undergone surgery in the past 4 months.
Ethics oversight	According to French Public Health Law (art L 1121-1-1, art L 1121-1-2), written consent and IRB approval are not required for human non-interventional studies.

Note that full information on the approval of the study protocol must also be provided in the manuscript.

ChIP-seq

Data deposition

☒ Confirm that both raw and final processed data have been deposited in a public database such as GEO.

☒ Confirm that you have deposited or provided access to graph files (e.g. BED files) for the called peaks.

Data access links<br>*May remain private before publication.*	Called peaks can be found in supplementary Table 3
Files in database submission	Bigwig files for input and ETV3 and ETV6 immunoprecipitation.
Genome browser session<br>(e.g. UCSC)	No longer applicable

Methodology

Replicates	2 biological replicates
Sequencing depth	Sequencing was performed in 1 sequencing unit of NovaSeq 6000 (Illumina) (100-nt593 length reads, paired end).
Antibodies	Chromatin was immunoprecipitated using antibodies against ETV3 (ref A303-737A, polyclonal, Bethyl) or ETV6 (clone R1092.1.1A9, CDI Laboratories Inc).
Peak calling parameters	Peak calling was performed with MACS2 using default parameters for each donor and corresponding input. Final peaks were obtained by intersect both donors using BEDTools with the parameter "-f 0.5". Peak assignment was performed on the intersect bed file using the annotatePeaks function of HOMER, using the genome "hg38" as reference.
Data quality	*Describe the methods used to ensure data quality in full detail, including how many peaks are at FDR 5% and above 5-fold enrichment.*
Software	*Describe the software used to collect and analyze the ChIP-seq data. For custom code that has been deposited into a community repository, provide accession details.*

Flow Cytometry

Plots

Confirm that:

☒ The axis labels state the marker and fluorochrome used (e.g. CD4-FITC).

☒ The axis scales are clearly visible. Include numbers along axes only for bottom left plot of group (a 'group' is an analysis of identical markers).

☒ All plots are contour plots with outliers or pseudocolor plots.

☒ A numerical value for number of cells or percentage (with statistics) is provided.

Methodology

Sample preparation	For human cultured cells, cells were stained in PBS containing 0.5% human AB serum and 2mM EDTA. For mouse cells, cells were stained in PBS containing BSA 0.5% and 2mM EDTA .
Instrument	Data was acquired on FacsVerse instrument (BD Biosciences) or MACSQuant (Miltenyi) instrument, or ZE5 flow cytometer (Bio-Rad). For imaging flow cytometry, cells were acquired on Amnis ImageStream instrument (Luminex).
Software	Flow cytometry data was analyzed with FlowJo v10 (FlowJo LLC).
Cell population abundance	Cell population abundance was calculated using FlowJo and is indicated for each sample.

Gating strategy

Gating strategies are described in supplementary figures. Single cells were gated based on SSC and FSC parameters, then gated on live cells.

☒ Tick this box to confirm that a figure exemplifying the gating strategy is provided in the Supplementary Information.
