# Supplementary figures and images for: ETV3 and ETV6 enable monocyte differentiation into dendritic cells by repressing macrophage fate commitment
Source: Nat Immunol. 2022 Dec 21;24(1):84–95. doi: 10.1038/s41590-022-01374-0 (PMC9810530; doi:10.1038/s41590-022-01374-0)

Fig1b left

GP96

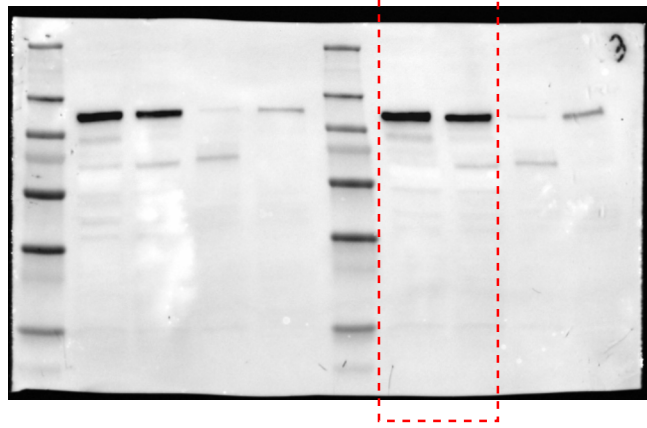

ETV3

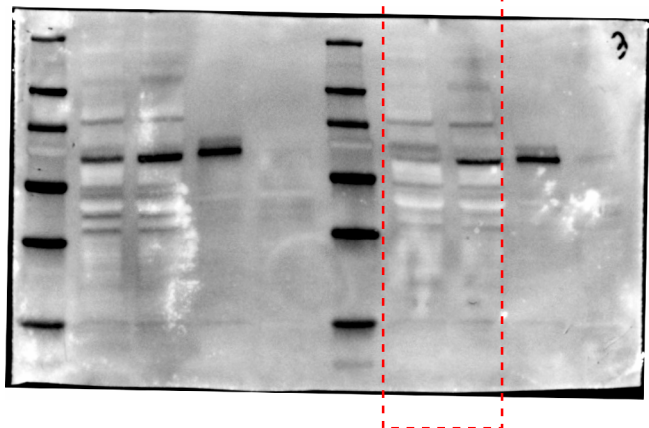

Fig1b right

GP96

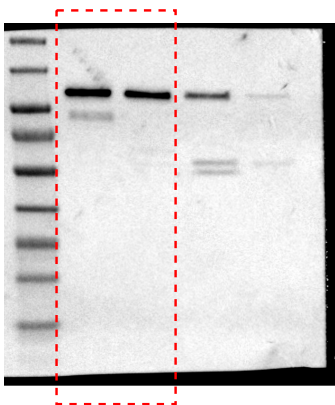

ETV6

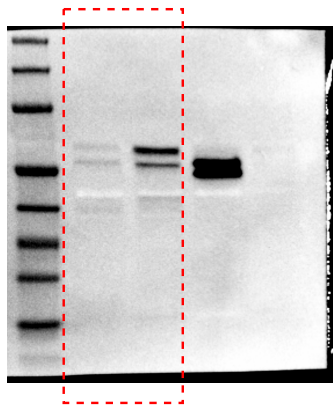

Fig1c

Actin

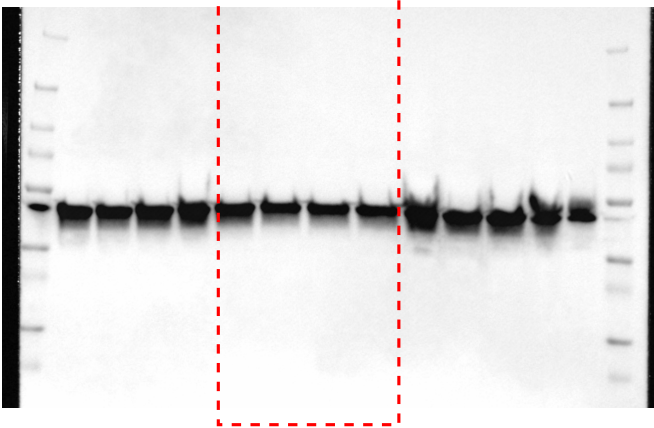

ETV3

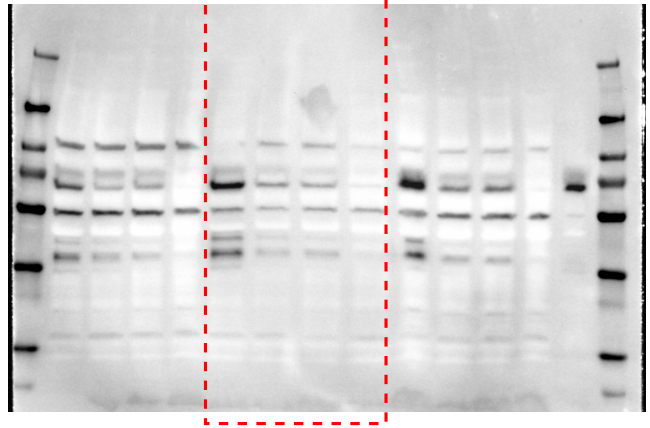

Fig1e

Actin

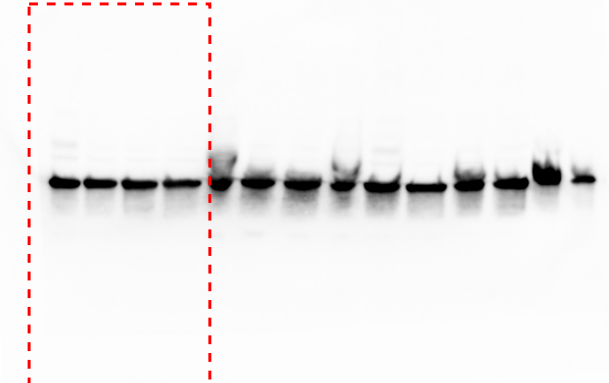

ETV6

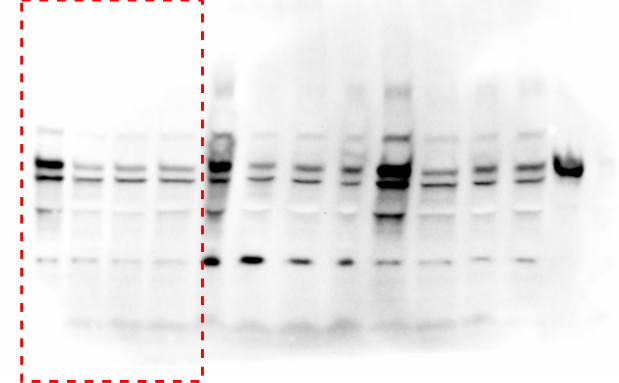

Supplement: Source Data Fig. 1 — Uncropped immunoblots from Fig. 1. The parts of the gels that were used for the figure panels are highlighted with a red box. [file 41590_2022_1374_MOESM6_ESM.pdf]

Fig4h

GP96

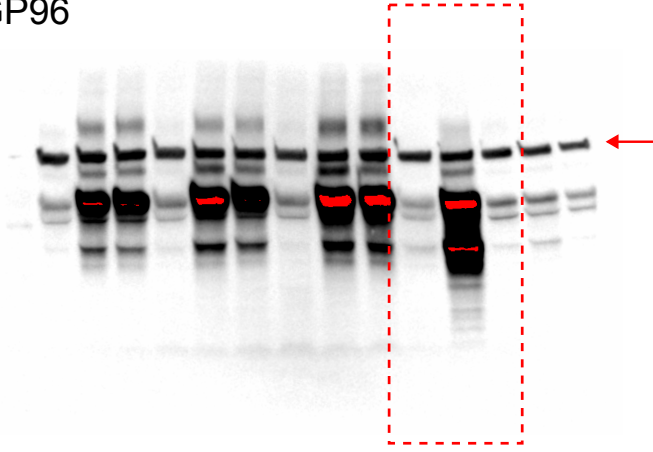

ETV6

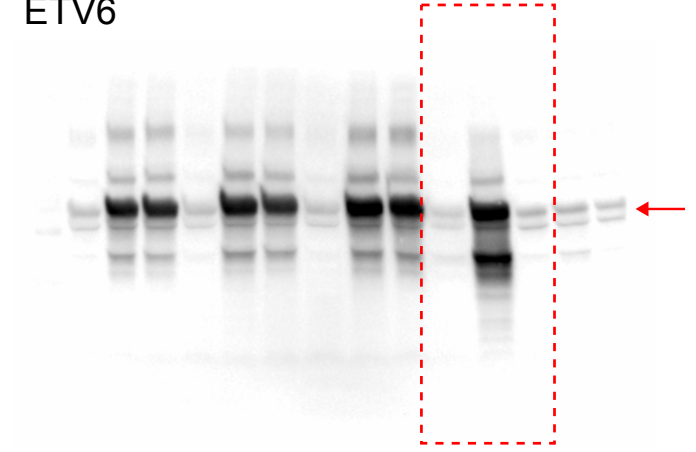

Supplement: Source Data Fig. 4 — Uncropped immunmoblots from Fig. 4. The parts of the gels that were used for the figure panels are highlighted with a red box. [file 41590_2022_1374_MOESM7_ESM.pdf]
